# Coronary artery mechanics induces human saphenous vein remodelling *via* recruitment of adventitial myofibroblast-like cells mediated by Thrombospondin-1

**DOI:** 10.7150/thno.40595

**Published:** 2020-02-03

**Authors:** Gloria Garoffolo, Matthijs S. Ruiter, Marco Piola, Maura Brioschi, Anita C. Thomas, Marco Agrifoglio, Gianluca Polvani, Lorenzo Coppadoro, Stefano Zoli, Claudio Saccu, Gaia Spinetti, Cristina Banfi, Gianfranco B. Fiore, Paolo Madeddu, Monica Soncini, Maurizio Pesce

**Affiliations:** 1 Unità di Ingegneria Tissutale Cardiovascolare; Centro Cardiologico Monzino, IRCCS; Milan, Italy; 2 PhD Program in Translational and Molecular Medicine - DIMET; Università di Milano - Bicocca, Milan, Italy; 3 Dipartimento di Elettronica, Informazione e Bioingegneria; Politecnico di Milano; Milan, Italy; 4 Unità di Proteomica; Centro Cardiologico Monzino, IRCCS, Milan, Italy; 5 Bristol Heart Institute, University of Bristol; Bristol, UK; 6 Dipartimento di Scienze Cliniche e di Comunità; Università di Milano, Milan, Italy; Centro Cardiologico Monzino, IRCCS, Milan, Italy; 7 Unità di Chirurgia Vascolare, Centro Cardiologico Monzino, IRCCS; Milan, Italy; 8 IRCCS Multimedica, Milan, Italy

**Keywords:** coronary artery bypass grafting, vein graft disease, mechanotransduction, Thrombospondin-1, arterialization

## Abstract

**Rationale**: Despite the preferred application of arterial conduits, the greater saphenous vein (SV) remains indispensable for coronary bypass grafting (CABG), especially in multi-vessel coronary artery disease (CAD). The objective of the present work was to address the role of mechanical forces in the activation of maladaptive vein bypass remodeling, a process determining progressive occlusion and recurrence of ischemic heart disease.

**Methods**: We employed a custom bioreactor to mimic the coronary shear and wall mechanics in human SV vascular conduits and reproduce experimentally the biomechanical conditions of coronary grafting and analyzed vein remodeling process by histology, histochemistry and immunofluorescence. We also subjected vein-derived cells to cyclic uniaxial mechanical stimulation in culture, followed by phenotypic and molecular characterization using RNA and proteomic methods. We finally validated our results *in vitro* and using a model of SV carotid interposition in pigs.

**Results**: Exposure to pulsatile flow determined a remodeling process of the vascular wall involving reduction in media thickness. Smooth muscle cells (SMCs) underwent conversion from contractile to synthetic phenotype. A time-dependent increase in proliferating cells expressing mesenchymal (CD44) and early SMC (SM22α) markers, apparently recruited from the SV adventitia, was observed especially in CABG-stimulated vessels. Mechanically stimulated SMCs underwent transition from contractile to synthetic phenotype. MALDI-TOF-based secretome analysis revealed a consistent release of Thrombospondin-1 (TSP-1), a matricellular protein involved in TGF-β-dependent signaling. TSP-1 had a direct chemotactic effect on SV adventitia resident progenitors (SVPs); this effects was inhibited by blocking TSP-1 receptor CD47. The involvement of TSP-1 in adventitial progenitor cells differentiation and graft intima hyperplasia was finally contextualized in the TGF-β-dependent pathway, and validated in a saphenous vein into carotid interposition pig model.

**Conclusions**: Our results provide the evidence of a matricellular mechanism involved in the human vein arterialization process controlled by alterations in tissue mechanics, and open the way to novel potential strategies to block VGD progression based on targeting cell mechanosensing-related effectors.

## Introduction

Coronary artery bypass grafting is used in cardiac surgery for more than 50 years to combat the consequences of coronary artery disease 1-3, a pathology with a wide incidence in Western world (> 1000 cases/ 10^6^ adult people) and rapidly increasing in emerging countries - e.g. China 4. The most represented coronary-compatible vessels used in CABG are the internal mammary artery (IMA), the radial artery (RA) and the SV. The patency of arterial grafts is relatively well preserved, while the SV conduits are subject to intima hyperplasia determining progressive graft occlusion. In a high percentage of cases, this requires re-hospitalization with graft stenting and, ultimately, re-intervention 1, 2. More in details, early vein graft failure due to acute thrombosis occurs in as many as 18% of cases. Intermediate graft failure (up to 2 years after surgery), and late graft failure (> 2 years after surgery), occurs in 20% to 50% of cases at 5 years. Finally, by 10 years after surgery, 40% of grafts result completely blocked and a further 30% have a compromised flow. Even if early remodelling of the vein is predictive for later graft patency, the aetiology of long-term failure is still poorly understood 5. Initially, both the surgical procedure and exposure to high flow and pressure compromise the endothelial layer, which induce SMCs proliferation 6, 7, changes in matrix composition, and thickening/stiffening of the vessel wall. This limits the vein's adaptability to the arterial circulation and ultimately leads to clinically apparent stenosis 8. Animal models revealed that occlusion of autologous SV grafts is a consequence of neointima formation, driven by proliferation and migration of SMCs and possibly of adventitial progenitors into the intima 9, inducing extracellular matrix (ECM) deposition, and formation of a thick neointima 10.

The role of mechanical forces in the progression of graft failure has been recognized, although the nature of cell-based mechanosensing in the vascular tissue remains unclear due the difficulty of decoupling distinct components *in vivo* 11, 12. In fact, the existing studies have not yielded conclusive results on the effect of mechanical stimulation on SMC growth and phenotype, with results depending on direction, frequency, duration and modality of the stimulus, but also on the origin and initial phenotype of the SMCs. For example, cyclic strain of human SV-derived SMCs resulted in increased DNA synthesis and cell number with a decrease in smooth muscle α-actin (αSMA) 13-15, while cells of arterial origin showed opposite effects 16, 17. *Ex vivo* vessel culture systems (EVCSs) and bioreactors to stimulate cells mechanically offer the unique possibility to investigate the effects of isolated or combined mechanical stimuli under well-controlled and reproducible biomechanical and/or metabolic conditions in human large vessels. In this framework, the aim of the present investigation was to characterize the effect of coronary mechanical conditions on molecular programming of vein graft disease using an integrated tissue/cell biomechanical approach.

## Methods

Extended Methods are provided in the Methods section in the online only Data Supplement.

### Ethics

The experimental investigation on human-derived tissues and cells was approved by the local ethical Committee at Centro Cardiologico Monzino, IRCCS. Patients were required to sign an informed consent. The use of human material was done in compliance with the Declaration of Helsinki. The main patient characteristics are shown in Table S1. Arteriovenous bypass procedures in pigs were performed in female Landrace or Large White/ Landrace crossbred pigs weighing 20 to 30 kg. All animals received humane care in accordance with the Home Office Animals (Scientific Procedures) Act of 1986 and the Guide for the Care and Use of Laboratory Animals published by the US National Institutes of Health (NIH Publication No. 85- 23, revised 1996). For surgery, anaesthesia was induced with a single dose of intramuscular ketamine into the neck (0.1 mg/Kg ketamine: Ketaset Injection Fort Dodge Animal Health Ltd, Southampton, UK). After endotracheal intubation, anaesthesia was maintained using 2-3% halothane and oxygen, the animals ventilating spontaneously throughout. Animals were euthanized with 100 mg/Kg intracardiac injection of pentobarbitone in a single dose (Euthatal; 200 mg/mL pentobarbital sodium, J.M. Loveridge Plc, Southampton, UK).

### Tissue/cells mechanical stimulations

Mechanical stimulations of SV grafts were performed using a custom-made bioreactor 18 tailored to reproduce the coronary mechanics. Cell straining was performed with Flex-Cell system. Mechanical stimulation times ranged from 7 to 14 days for SV grafts and from 1 to 3 days for cells.

### Tissue/cells analyses

After mechanical stimulation, tissues, cells and culture supernatants were prepared and appropriately processed for histological, immunohistochemical, immunofluorescence, protein/secretome and RNA analyses, as already published 19, and described in the extended online methods.

### *In vitro* cell culture

Isolation of cells for *in vitro* experiments was performed as previously described 20, 21, using immunomagnetic and/or plastic adherence selection. Migration experiments were performed using Transwell-based assays followed by quantification of Crystal-Violet cell staining.

### Molecular analyses

Tissue/cells RNA and protein content was analysed by Q-RT-PCR and Western blotting performed with protocols already published 19, while the secretome analysis was conducted using a MALDI-TOF methods as already described 22.

### Data representation and statistical analyses

In all graphs throughout the manuscript, data were plotted as mean ± standard error using GraphPad Prism 7. A *P* value < 0.05 was considered significant. The type of statistical test employed for data comparison is specified in figure legends. As a general rule, comparisons between two independent samples were performed by unpaired/paired t-test (two-tails), while for comparisons between 3 or more groups we adopted one-way ANOVA with post-hoc comparisons tests. The specification of the number of independent samples included in the analyses and the type of statistical tests used to compare data are specified in the legends to figures. Further details about data processing are provided in the online methods section.

## Results

### CABG-like hemodynamics induces consistent remodeling of human SV wall associated to SMCs phenotypic switching

**Figure 1:**
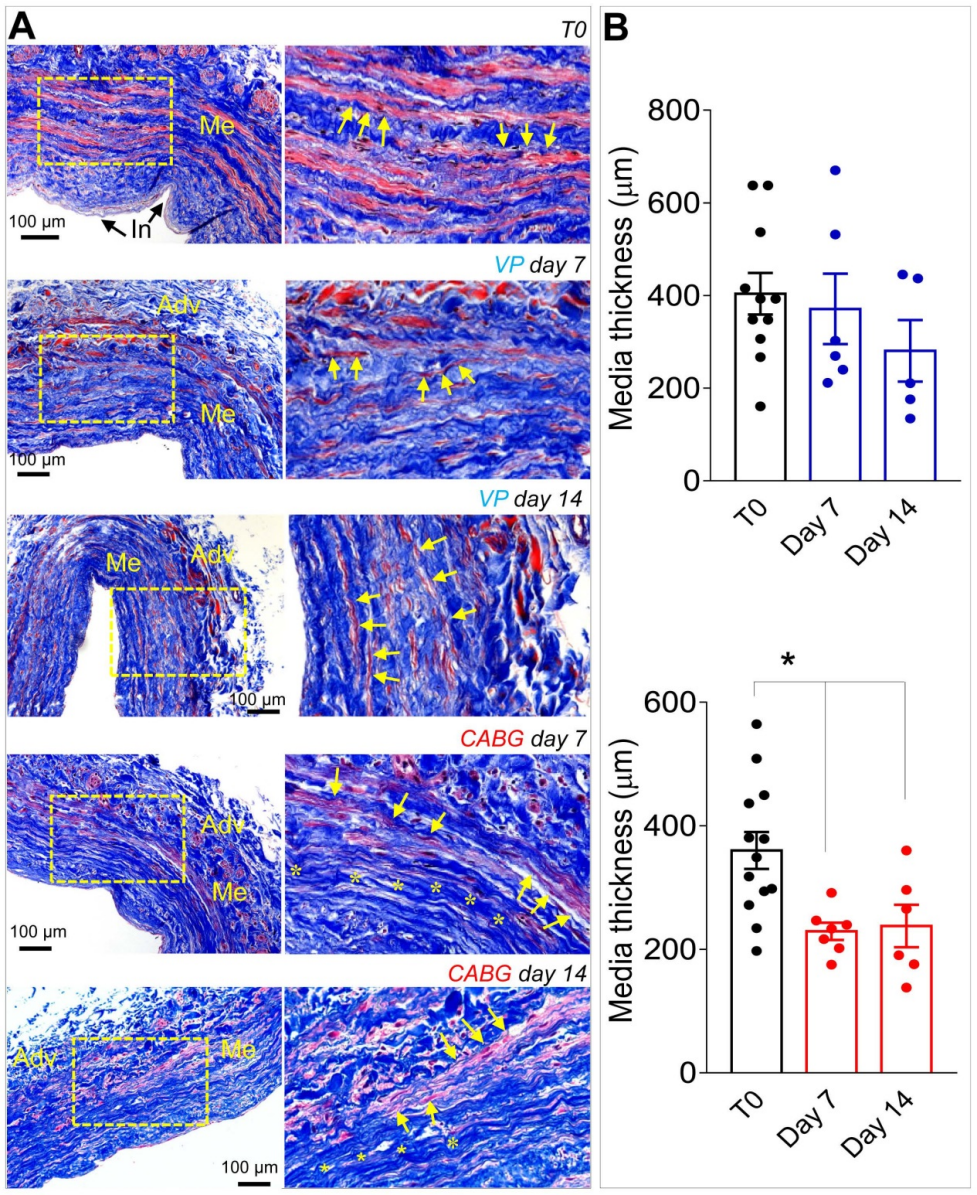
(A) Low and high magnifications (zones enclosed in the yellow areas) of transversal sections of native SVs (T0) and SVs exposed to venous perfusion (VP) or coronary flow (CABG) for 7 and 14 days, stained with Masson's trichrome. While in VP samples, the circumferentially arranged SMCs bundles (characterized by the red staining, yellow arrows) are present throughout the culturing period, in SVs exposed to CABG mechanics, part of these bundles disappeared (areas stained in blue, asterisks) leaving zones rich in collagen and deprived of cells. This suggests that circumferential SMCs bundles are a specific target of coronary flow mechanics. (B) Quantification of media thickness, confirmed a major effect of the CABG stimulation on SV wall remodeling. * indicate P < 0.05 by one-way ANOVA with Newman-Keuls post-hoc. Bar graphs represent mean and SE. Me = Media; Adv = Adventitia: In = Intima.

Our previous contributions showed that application of an 80-120 mmHg pulsatile pressure regimen determined a change in the SV structure at 7 days, consisting of a significant thinning of the vessel wall, and elevation in cell death by apoptosis and enhanced proliferation 18, 19. These changes were less evident in SVs exposed to venous flow (VP), characterized by a constant low pressure (5 mmHg). Confirming these findings, morphometric analyses of SVs treated with coronary flow showed a significant thinning of the medial layer (Figure 1A-B). In order to demonstrate the specific effects of mechanical forces on intima thickening, we compared results obtained in the presence of venous or coronary flow/pressure patterns with a conventional vein 'rings' culturing model^23^. Figure S1 and S2 show results of this experiment, which demonstrate that in the presence of coronary flow-pressure pattern intima thickening does not occur, at least at the considered time points.

**Figure 2:**
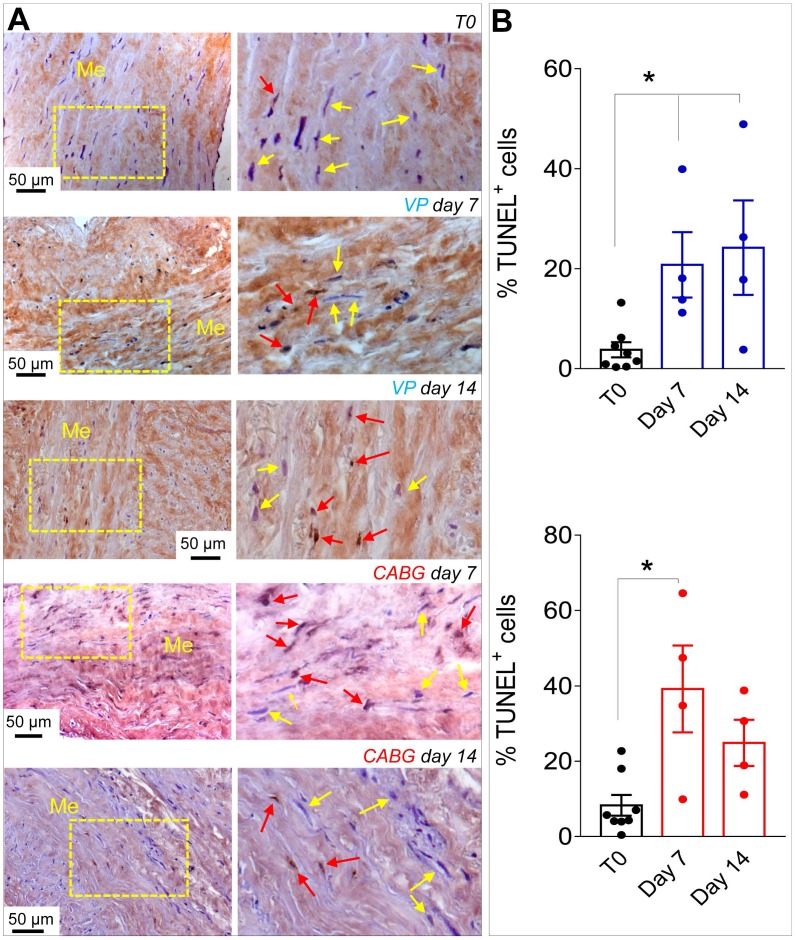
(A) Low and high magnifications (zones enclosed in the yellow areas) of transversal sections of native SVs (T0) and SVs exposed to venous perfusion (VP) or coronary flow (CABG) for 7 and 14 days stained with TUNEL assay for detection of apoptotic cells. Yellow arrows indicate TUNEL^-^ cells while red arrows indicate TUNEL^+^ apoptotic cells. (B) Apoptosis quantification in the SV media at the two time points. * indicate P < 0.05 by one-way ANOVA with Newman-Keuls post-hoc. Bar graphs represent mean and SE. Me = Media.

**Figure 3:**
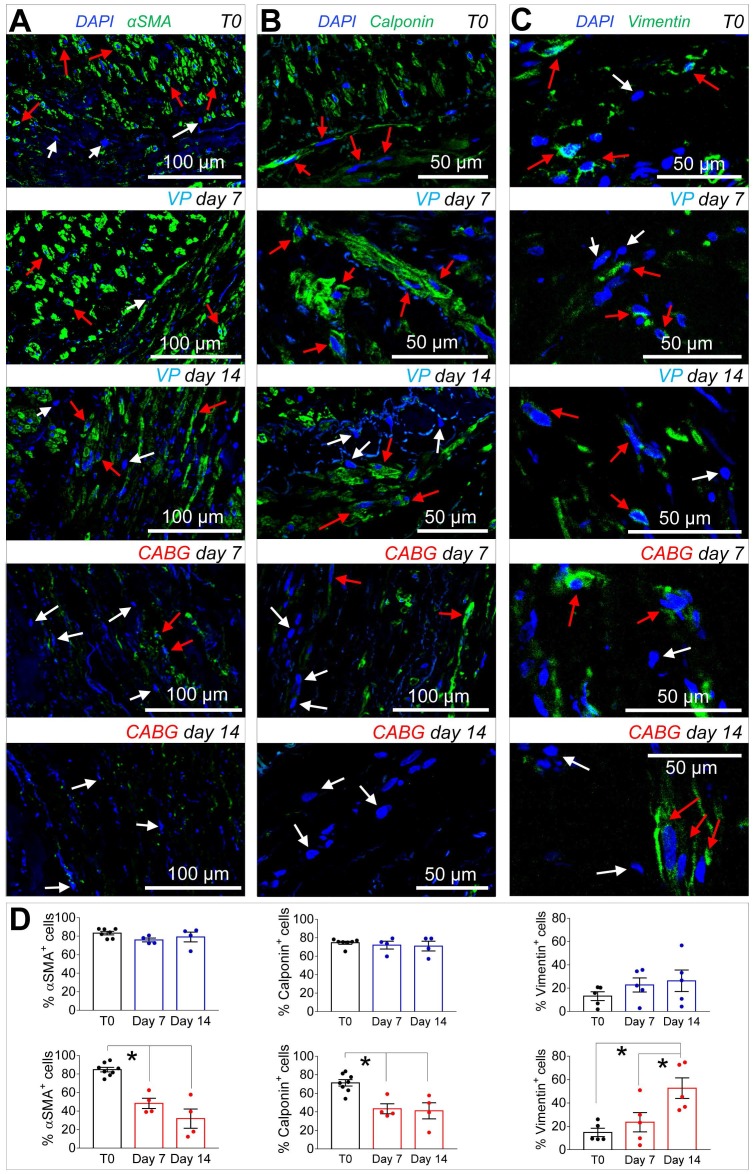
(A-C) Confocal microscopy images of transversal sections of native SVs (T0) and SVs exposed to venous perfusion (VP) or coronary flow (CABG) for 7 and 14 days stained with antibodies recognizing contractile (αSMA, Calponin) and secretory (Vimentin) SMCs markers. Red arrows indicate cells expressing the indicated markers, while the cells indicated by white arrows are marker^-^ cells. (D) Quantification of the marker^+^ cells expressed as a percentage of the total nuclei count present in the SV medial layer. Data are presented per treatment groups with blue bars indicating VP and red bars indicating CABG. * indicate *P* < 0.05 by one-way ANOVA with Newman-Keuls post-*hoc* comparison. Bar graphs represent mean and SE.

Since *ex vivo* cultured SVs exhibited a similarly elevated cellular apoptosis (Figure 2A-B), we investigated whether application of a coronary-like flow pattern affected the phenotype of the surviving cells. To this aim, we performed IF staining with antibodies recognizing SMCs contractile markers αSMA (Figure 3A)/Calponin (Figure 3B), and the synthetic SMCs marker Vimentin (Figure 3C). Cells quantification (Figure 3D) demonstrated a clear reduction in the percentage of αSMA^+^ and Calp^+^ cells in SV conduits stimulated with coronary flow/ pressure pattern, and a consistent increase in cells with synthetic characteristics, suggesting a SMCs phenotypic transition.

### Coronary mechanics induces recruitment and proliferation of CD44^+^ cells in SV medial layer

**Figure 4:**
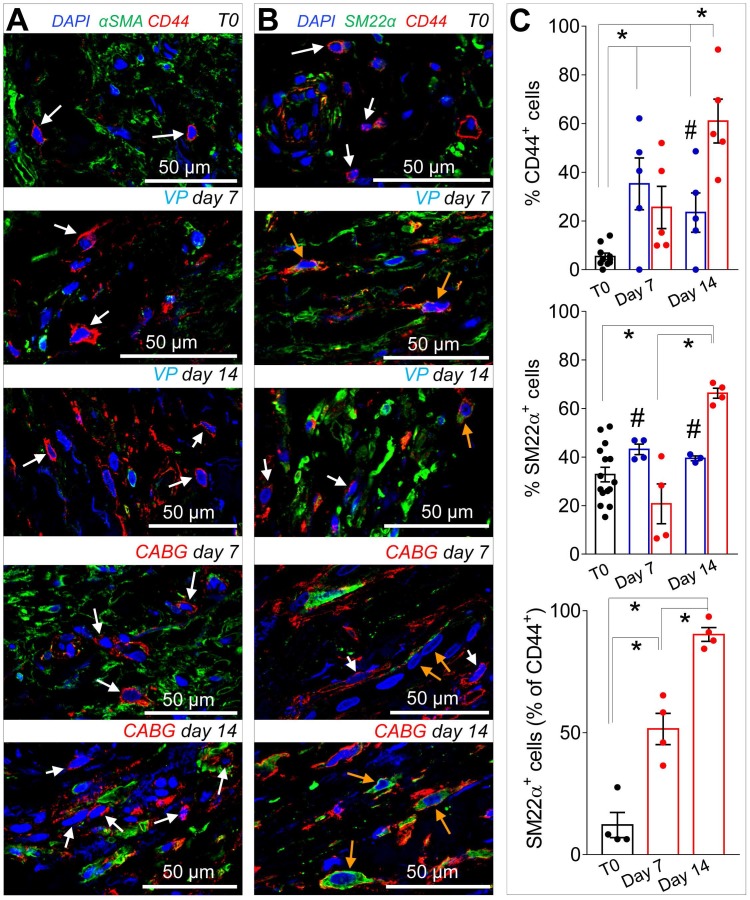
(A-B) Low and high magnifications of confocal images of SV tissue sections stained with CD44 with αSMA, (A) or SM22α (B) antibodies. While expression of αSMA was independent of that of CD44 (white arrows) a staining overlapping of CD44^+^ and SM22α^+^ (orange arrows) was observed especially in CABG samples at day 14 of stimulation. (**C**) Quantification of single marker^+^ cells or SM22α^+^/CD44^+^ cells in the medial layer of SV conduits exposed to venous perfusion or coronary flow revealed a sharp increase in double positive cells in the CABG conditions. * indicate *P* < 0.05 by one-way ANOVA with Newman-Keuls comparison tests; # indicate P < 0.05 by unpaired Student's tests at the corresponding time points between the two treatments. Bar graphs represent mean and SE.

**Figure 5:**
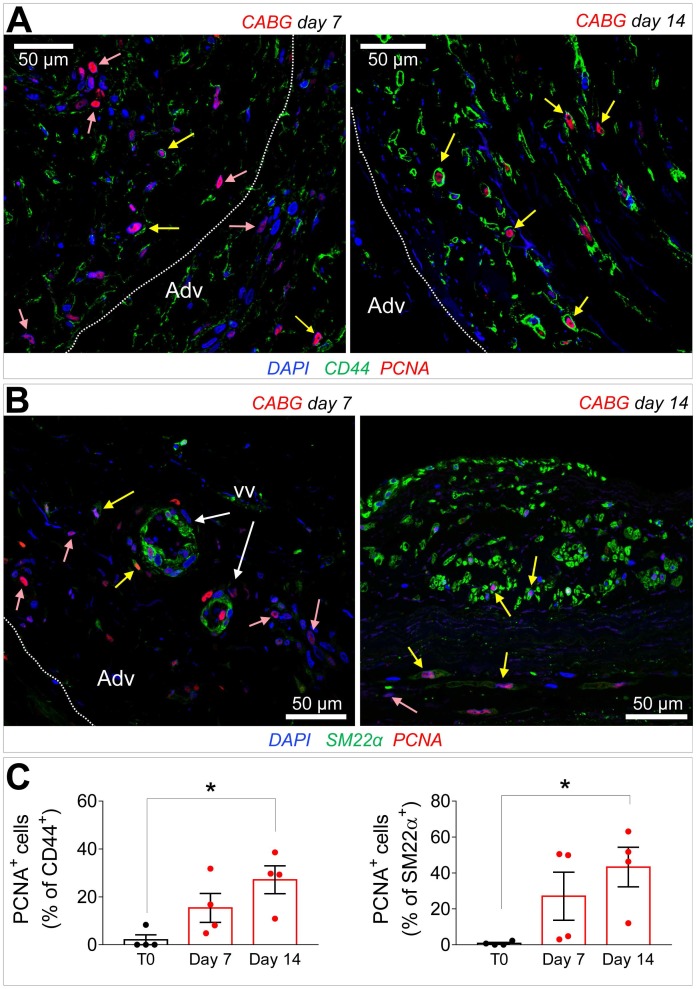
(A-B) Immunofluorescence staining of CABG-treated SV conduits sections with PCNA marker in conjunction with CD44 (A) or SM22α (B). PCNA^+^ cells co-expressing (yellow arrows) or not co-expressing (rose arrows) the mesenchymal and the SMC markers were localized in the adventitia (Adv) at 7 days also in association with the *vasa vasorum* (VV) Dotted lines indicate the position of the external elastic lamina. (C) Quantification of CD44^+^/PCNA^+^ and SM22α^+^/PCNA^+^ cells in the medial layer. * indicate *P* < 0.05 by one-way ANOVA with Dunnett's post hoc comparison test. Bar graphs represent mean and SE of observations.

Recruitment of cells from the vascular adventitia has been identified as a key early event in VGD setting in animal vein arterialization models 24. Furthermore, microscopic observations performed in explanted CABGs or SV grafts exposed *ex vivo* to *trans*-wall hypoxia gradients showed an enhanced growth of adventitia vessels 25. Since CD44 marker expression has been associated to myofibroblast programming of stromal cells in fibrotic diseases 26, 27, we investigated the expression of this marker after culturing SV grafts. In order to assess if cells expressing CD44 also expressed SMCs markers, we performed co-staining with anti-αSMA and -SM22α specific antibodies in control and *ex vivo* cultured SVs. As shown in Figure 4A-B very few CD44^+^ cells were present in the media of the vessels before the beginning of the culture. However, these cells increased time-dependently, in particular in CABG samples, where they exhibited a clear co-staining with SM22a. Quantification of cells confirmed that CD44^+^ and SM22α^+^ cell percentages increased under either VP or CABG stimulation at day 7, and continued to rise in CABG-stimulated vessels at day 14 (Figure 4C). The presence of CD44^+^/SM22α^+^ cells in CABG-treated SVs exhibited, finally, a substantial and constant increase (Figure 4C). Since the majority of CD44^+^ and SM22α^+^ cells at the beginning of the culture were confined in the adventitia in close association with the *vasa vasorum* (Figure S3), this suggests that coronary flow/pressure pattern activates adventitial cells expressing myofibroblasts/immature SMCs markers. In keeping with this conclusion, increase in CD44^+^/SM22α^+^ cells in CABG-stimulated grafts was associated to an elevated proliferation level (Figure 5A-C), which was more pronounced initially in the adventitia (in particular in the *vasa vasorum* region), and later in the media, as detected by expression of PCNA marker.

### Thrombospondin-1 is a mechanically regulated factor in the SV wall associated to SMCs switching from contractile to synthetic phenotype

**Figure 6:**
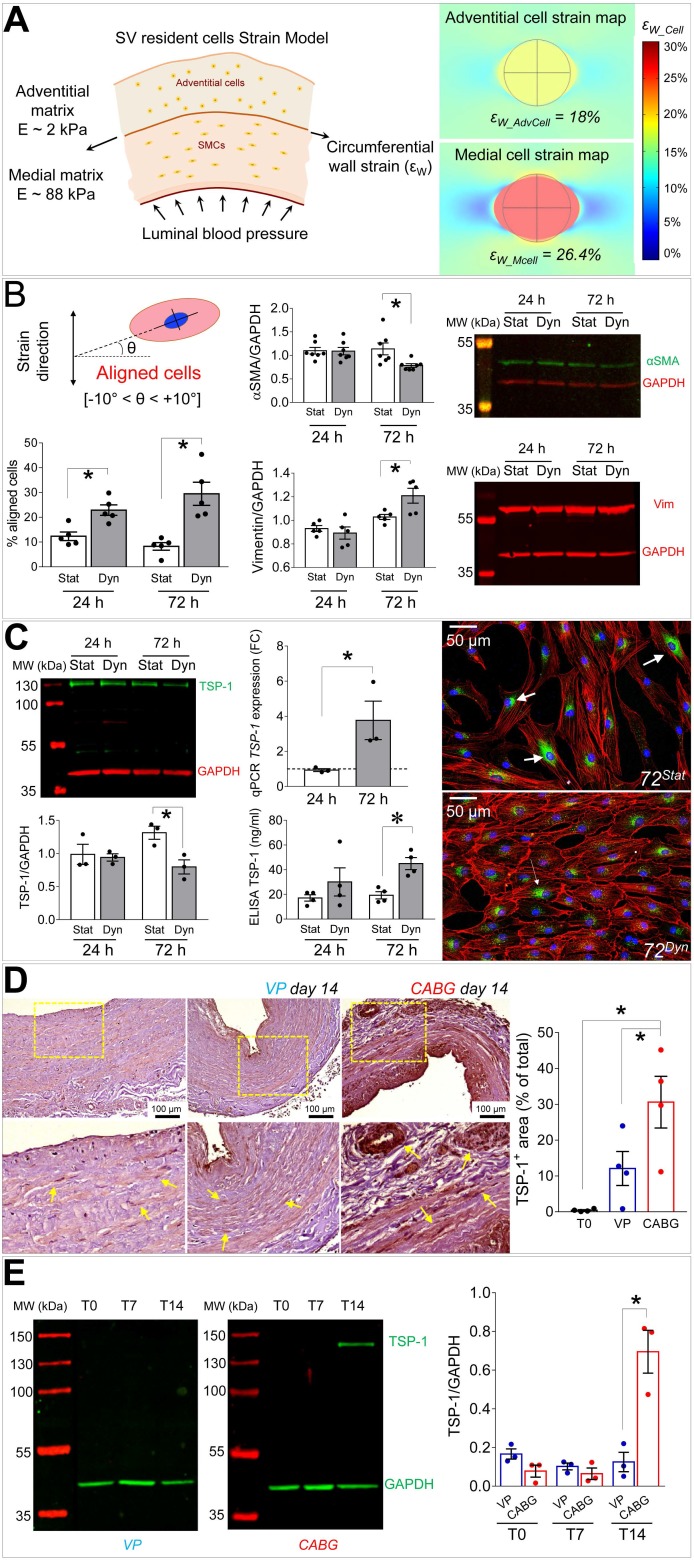
(A) On the left side of the panel, a schematic of the structural components, the stiffness characteristics (expressed as Young's modulus, E [kPa]) and the circumferential wall strain (ε_W_) employed to compute cell stretching experienced by cells in the adventitial and in the medial layer when coronary blood pressure is applied. On the right side of the panel it is represented the predicted cell deformation maps in the medial (ε_W_Mcell_) and adventitial (ε_W_AdvCell_) layers, as a result of the circumferential wall strain. (B) Results of 10% cyclic stretching on orientation of SV-SMCs (see scheme showing the procedure adopted to calculate cellular orientation above the bar graph on the left); modulation of αSMA and Vimentin by Western blot analysis (right). (C) Western blotting and IF (left side and right side, respectively) of TSP-1 into mechanically stimulated cells; ELISA tests of TSP-1 release in the medium. *TSP-1* gene expression analysis by Q-RT-PCR (central part of the panel). (D) TSP-1 immunohistochemistry in VP and CABG-stimulated SV samples. Statistical analysis indicated a significant difference in signal intensity at day 14 in CABG (red bars) *vs.* VP (blue bars) and T0 (white bars) samples. (E) Western blotting analysis of whole protein extracts from SV samples. As shown in the quantification graph, CABG-treated samples at day 14 (red bars) underwent a dramatic upregulation of TSP-1 compared with VP samples (blue bars) and earlier CABG time points, or controls. * indicate *P* < 0.05 by, (B, C) paired t-test, (D) one-way ANOVA with Newman-Keuls multiple comparison post-*hoc* test and unpaired t-test (E). Bar graphs represent mean and SE of observations.

Mechanical forces exerted by the counter-pulsed coronary flow on vein wall expose vein-resident cells to a high level of strain, whose distribution is modified compared to the natural venous perfusion 12. We then modelled *in silico* the level of cell deformation associated to the circumferential strain of the two major SV layers occurring in the presence of coronary flow mechanics. We were interested in this strain component considering the orientation of the circumferentially arranged SMCs bundles, which appeared the most affected structures in the media of CABG-stimulated SV conduits (Figures 1 and 3). As shown in Figure 6A, the model predicted a more pronounced strain in the stiffer media (~ 26% elongation), and a lower strain value (~ 18% elongation) in the softer adventitia. Based on this evaluation, we decided to investigate the effects of uniaxial cell deformation on SMCs isolated from human SVs (Figure S4) using an *in vitro* cyclic cell strain setting. Figures S5 and 6B show the results of 24 and 72 h SMCs mechanical stimulation, which determined a significant reorientation of the cells. Under these conditions, these cells downregulated the contractile phenotype marker αSMA, and upregulated the levels of Vimentin (Figure 6B). They also exhibited a substantial rearrangement of the contractile cytoskeleton organization, which acquired an arrangement consistent with the synthetic phenotype 21 (Figure S6).

In order to find a relationship between the phenotypic switch occurring in SMCs and the observed activation and presence of CD44^+^ cells, we performed a secretome analysis of proteins released in the culture medium by mechanically strained SMCs using a mass spectrometry-based approach. This indicated the matricellular protein Thrombospondin-1 (TSP-1) 28 as a factor released at high levels both at 24 and 72 h of mechanical stimulation (Table S2). Mass-spec data were validated in independent biological replicates using IF and ELISA tests, which confirmed TSP-1 release from SMCs (Figure 6C). Interestingly, Western analysis of the protein extracts revealed a decrease of the intracellular content of TSP-1 in mechanically stimulated SMCs (Figure 6C). Altogether these data indicate that mechanical strain induces release of TSP-1 from intracellular stores and a concomitant upregulation at transcriptional level, thus identifying *TSP-1* as mechano-responsive gene. Finally, we performed immunohistochemistry and Western blotting analyses on tissue sections and protein extracts of samples treated with VP or CABG flow. This clearly showed a specific elevation of TSP-1 in the intima and media compartments of CABG-stimulated *vs.* VP and control SVs (Figure 6D-E). Immunofluorescence staining with anti-TSP-1 and -SM22α antibodies indicated the presence of TSP-1^+^ cells with or without co-expression of the early SMCs marker in CABG-treated samples (Figure S8).

### TSP-1 induces migration of SV adventitial progenitor cells

**Figure 7:**
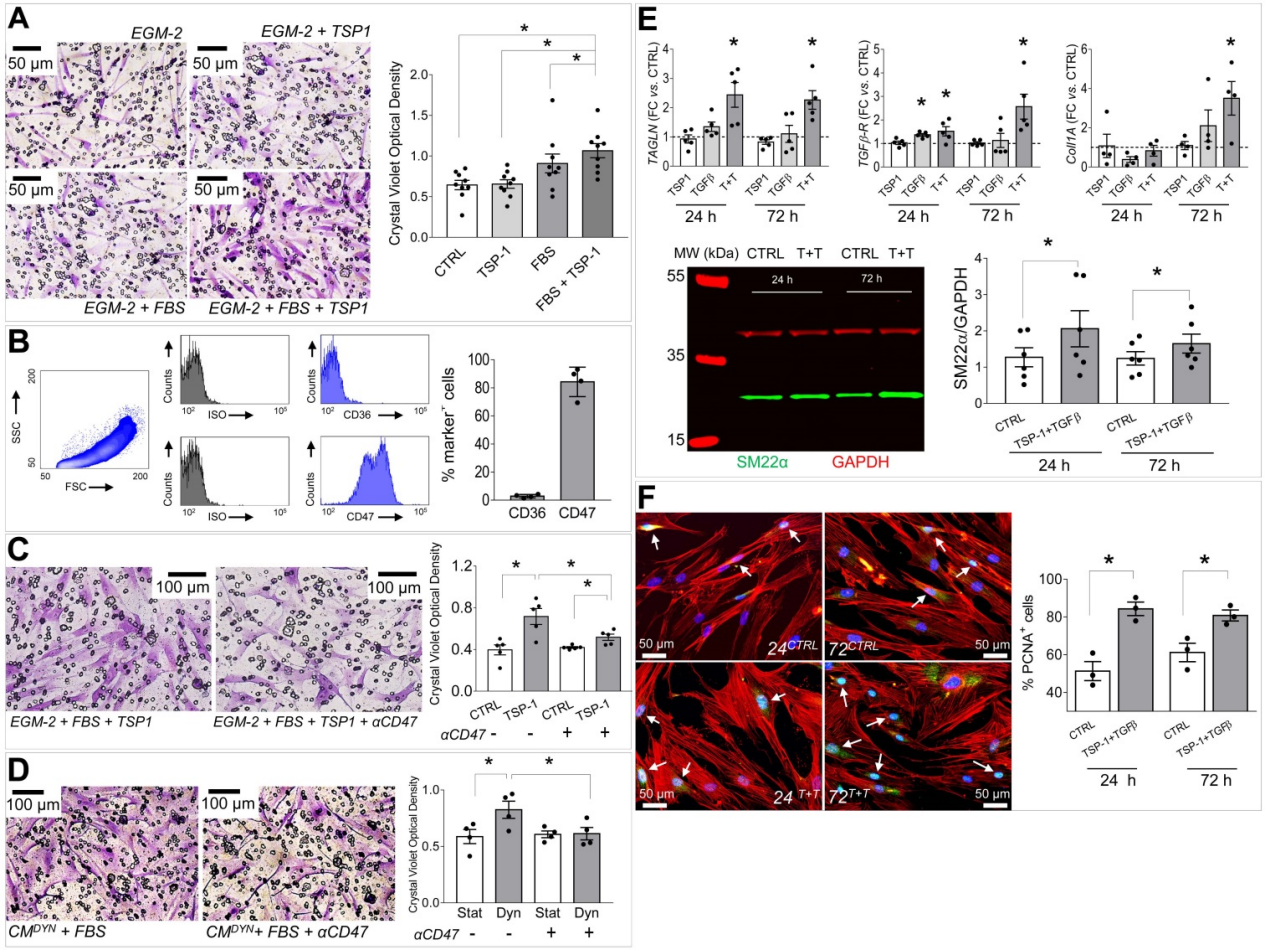
(A) SVPs transwell migration assay; micrographs show the cells that migrated through the membrane pores stained with Crystal Violet (purple color). Bar graph indicates quantification of the Crystal Violet optical density (see methods). * indicate *P* < 0.05 by one-way ANOVA (repeated measures) with Newman-Keuls multiple comparison post hoc test. (B) FACS analysis of the main TSP-1 receptors in human SVPs (n = 4). (C-D) Inhibition of SVPs migration against recombinant TSP-1 (C) and strained SMCs conditioned medium (D) by CD47 blocking antibody. * in both panels indicate *P* < 0.05 by paired student's t-test. (E) Effect of TSP-1/TGF-β treatment on SVPs phenotype. Q-RT-PCR analysis of *TAGLN* (SM22α), *TGFβR*, *Col1A* genes expression (upper). Data are represented as fold change (FC = 2^-ΔΔCT^) *vs.* untreated cells cultured for the same amount of time (dotted line). * indicate P < 0.05 by paired t-test performed on ΔCT values. Western blotting analysis of SM22α protein expression (lower). * indicates P < 0.05 by paired t-test. (F) Effect of TSP-1/TGF-β treatment on SVPs proliferation (PCNA immunofluorescence, green). * indicate *P* < 0.05 by paired t-test. Bar graphs represent mean and SE of observations.

The human SV adventitia contains progenitor cells with pericyte characteristics, the so-called saphenous vein progenitors (SVPs) 20. These cells are characterized by CD44 as well as other fibroblast/stromal cells markers (Figure S9) 26, and may represent a potential source of cells participating to SV graft pathologic evolution by differentiating into myofibroblasts and SMCs 12. In order to correlate the mechanical-dependent TSP-1 regulation in the SV wall and activation/recruitment of CD44^+^ cells, we performed migration assays in Transwells (Figure 7A). Results showed that TSP-1 elevated the migration of the cells compared to serum only. As established in literature, TSP-1 exerts its cellular functions through specific receptors 28. We therefore investigated the expression of TSP-1 receptors in SVPs using specific antibodies for CD36 and CD47, and this highlighted a high CD47 expression level (Figure 7B). To substantiate the role of TSP-1 on SVP migration, we treated cells with a blocking CD47 antibody in migration assays against the protein (Figure 7C) or the stretched SMCs conditioned medium (Figure 7D); in both cases, treatment with the antibody inhibited SVPs migration.

### Integration of mechanosensing- and humoral-driven pathways in pleiotropic SVPs responses in saphenous vein graft failure

**Figure 8:**
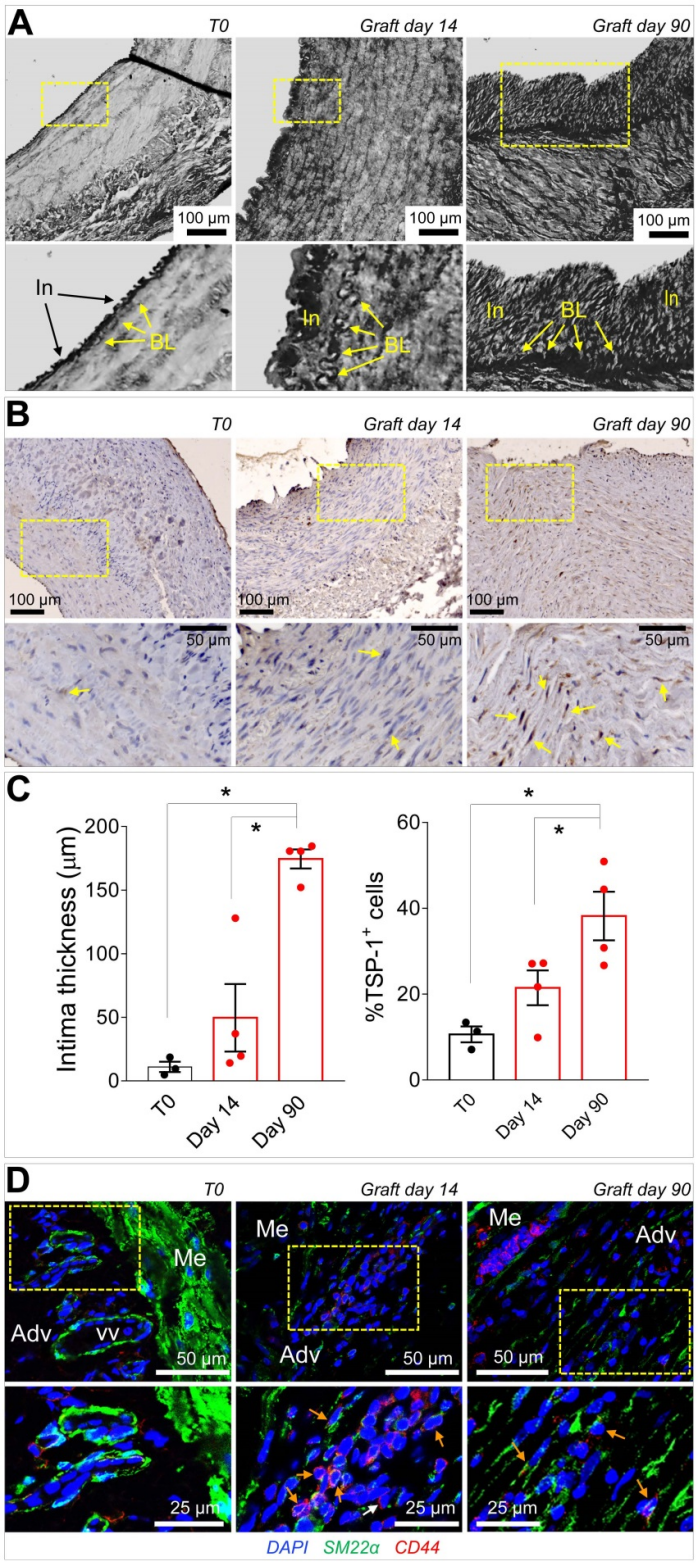
(A) Low and high magnifications (zones enclosed in the yellow areas) of transversal sections of porcine SV grafts before, and after 14 and 90 days *in vivo* arterialization. Yellow arrows indicate the intima basal lamina (BL). (B) Low and high magnifications (zones enclosed in the yellow areas) of section stained with anti TSP-1 antibody. Yellow arrows indicate TSP-1^+^ cells. (C) Quantification of intima thickening and of TSP-1^+^ cells. (D) Staining with antibodies directed against SM22α and CD44 of control (T0) and *in vivo* arterialized (day 14; day 90) SV in pigs. In the high magnifications on the bottom (correspondent to the zones enclosed in the yellow areas), orange arrows indicate cells expressing the two markers. * indicate P < 0.05 by one-way ANOVA with Newman-Keuls post-hoc test. Bar graphs represent mean and SE of observations.

Taken together, the previous data suggested that CD44^+^/SM22α^+^ cells growing in SV conduits exposed to coronary mechanics are actively recruited from the adventitia where they initially reside; conversion from contractile to synthetic SMCs due to circumferential strain component attracts these cells in the medial layer through secretion of TSP-1 and CD47-mediated chemotaxis. To get further insights in this process, we reasoned that transitioning from the adventitia to the media could expose cells to a combination of stimuli resulting from TSP-1 and classical vascular *pro*-remodeling factors, such as TGF-β 29. On the other hand, we already showed that arterial-mimicking pressure elevates the expression of TGF-β in the SV, and literature reported a crucial function of TSP-1 for TGF-β pathologic activation of cells with mesenchymal and smooth muscle cells phenotypes 30, 31. In order to substantiate this hypothesis, we stimulated SVPs with TGF-β (10 ng/mL) and TSP-1 (50 ng/mL), alone or in combination, followed by analysis of SM22α, Collagen1, TGF-βR, and cell proliferation. Results showed an increase in the expression level of the SMC/myofibroblast differentiation markers (Figure 7E-S10) and proliferation (Figure 7F-S11) at both times in cells treated with the factors combinations. For a final confirmation of TSP-1 expression in failing of arterialized veins, we performed immunostaining with TSP-1 antibodies in sections of SV grafts transplanted into carotids in pigs 32. As shown in Figure 8A-C, SV grafts underwent a significant thickening of the intima layer, which peaked at 90 days post-transplantation. Concurrently, an increased number of cells expressing TSP-1(Figure 8B-C) and of SM22α^+^/CD44^+^ cells (Figure 8D) was observed in the wall of the implanted SVs.

## Discussion

We and others have hypothesized an important role of hemodynamic forces in molecular programming of the SV graft disease 12. On the other hand, the lack of platforms mimicking the peculiar flow/pressure pattern existing in the coronary circulation has prevented the identification of molecular pathways connected to alterations of SV mechanics directly in human samples. In the present investigation we filled this gap using a platform customized to maintain vessel viability and reproducing the hemodynamic conditions of the coronary circulation 18. We found a major vessel remodeling effect consisting in, *i)* a substantial rearrangement of the intima and the media layers structure, *ii)* a major change in cell composition resulting from an early induction of apoptotic death in a significant portion of the SMCs, accompanied by a transition from a contractile to a synthetic phenotype of the surviving SMCs associated with TSP-1 release in the media, and *iii)* proliferation of a vessel-resident cell subset arising from the SV adventitia, characterized by expression of CD44 and SM22α, leading to a fibrotic-like response associated to TSP-1 release.

### Coronary mechanics induce SMCs phenotypic transition toward a synthetic phenotype and growth of a myofibroblast-like cell population in the SV media

The first questions that we aimed to resolve was to define the structural rearrangement of the vessel wall subjected to CABG conditions and the cellular dynamics occurring in consequence of the coronary stimulation *vs.* the venous flow/pressure pattern.

As shown in Figures 1, S1 and S2, under CABG conditions, the SV wall underwent a consistent thinning with reduction of the overall media thickness. Interestingly, the coronary-like stimulation was not associated to intima growth, while VP-stimulated vessels (analogous to the conventionally accepted SV culture method) exhibited significant intima thickening at day 14. This result, although contra-intuitive, is in line with observations performed in SV CABGs explanted *post mortem* showing narrowing of the vein wall and absence of intima hyperplasia at early times after implantation 33. It further supports the hypothesis, already made in two other previous publications from our groups 18, 25, that the SVs contain a certain degree of damage before the culture that depends on surgical manipulation (e.g. interruption of collateral vessels, partial removal of the adventitia). In support of this hypothesis is, finally, the finding that TUNEL staining showed a similar level of apoptotic cells, especially at 7 days of culture, irrespective of the flow pattern (Figure 2).

IF data indicated that coronary mechanics induced synthetic SMCs phenotype, with a decrease in αSMA and Calponin and a parallel increase in Vimentin in the media layer (Figure 3). This is consistent with observations performed in animal models, in which medial SMCs lose contractile markers in favor of a synthetic phenotype 34, 35. It is interesting to note that while disappearance of cells with contractile markers peaked at day 7, a consistent increase in Vimentin^+^ cells occurred at day 14 of CABG stimulation. Thus, the shift in SMCs phenotype in the media was likely an effect of the adaptation of the cells surviving the early apoptotic peak to the new mechanical conditions. This conclusion is in line with reported differences in biological responses of synthetic SMCs to mechanical strain compared to their contractile counterparts 36. The different response of these cells in the CABG mechanical condition may be finally part of the TSP-1-mediated pathway, given the reported protective effects from apoptosis of TSP-1/CD47 interactions in SMCs 37.

Our immunofluorescence analyses of the SV wall revealed that in CABG condition, the vascular wall was repopulated by cells expressing the mesenchymal marker CD44 and early SMC marker SM22α. Due to marker overlapping between these strictly associated cell types, and the absence of methods to perform lineage-restricted cell tracking in the human tissue, it is at present not possible to conclude definitively whether these cells derive from direct activation of pre-existing CD44^+^/SM22α^+^ SMCs or of progenitors with pericyte characteristics associated to *vasa vasorum*^38^. CD44 is, in fact, the ligand for hyaluronan, and it is involved in cell-cell/cell-matrix interactions in SMCs, myofibroblasts and inflammatory cells. This glycoprotein has been associated with SMCs differentiation 39, and high levels of CD44 have been associated with cell migration, injury-induced remodeling and fibrosis 27, 40. SM22α/Transgelin is a F-Actin associated protein expressed in smooth muscle cells and fibroblasts, whose control has been recently associated to cell mechano-transduction 41. Interestingly, in unstimulated samples SM22α^+^/CD44^+^ cells were found almost uniquely in the adventitia in the *vasa vasorum* region, from where they appeared to invade the media at the later time point, especially in CABG condition (Figure S3, Figure 5). This finding is suggestive of a two-step adventitial cells recruitment process consisting of, at first, activation in the adventitia and, thereafter, active migration/proliferation in the media. Although these results are in contrast with findings obtained by passive culturing of SV vein rings (where there is no intervention of mechanical forces, Figure S1-S2) 23, they confirm findings in real CABGs explanted *post mortem*, where no intima hyperplasia was observed up to one month after implantation 33 and previous results obtained in our laboratory showing a structural rearrangement of *vasa vasorum* in response to hypoxia or mechanical injuries 19, 25. Furthermore, they are in line with studies showing the contribution of adventitial progenitors to vein graft failure in animal models of vein arterialization 24.

### A matricellular pathway mediated by Thrombospondin-1 accounts for activation of resident myofibroblast-like cells in SVs exposed to coronary mechanics

Cell sensitivity to mechanical cues is becoming more and more relevant for the progression of chronic fibrotic diseases as a fundamental part of damage repair process 42, 43. Our *in silico* modelling of the cell strain in the two main layers of the SV wall predicted a particularly elevated level of mechanical stress for SMCs in the media (particularly those arranged in the circumferentially arranged bundles) (Figure 6). It was therefore crucial to expose these cells directly to cyclic mechanical stress *in vitro* and assess existence of signals secreted by these cells potentially involved in activation of adventitial resident cells. Given that *in vivo* mechanical stress sensed by these cells has a major uniaxial component 12, we performed a stimulation protocol along a unique direction with a 10% deformation level. Although this protocol led cells to experience a nominal deformation level lower than the maximal predicted by the model in the SV wall, we reasoned that mechanical forces in the real tissues is sensed by cells with a complex dynamics involving partial absorption by surrounding matrix due to its viscoelastic properties, while in the 2D condition cellular deformation occurs through direct transmission of elastic forces to cytoskeleton by the focal adhesion complexes 44. The choice of a mass spectrometry approach to analyse the secretome of strained SMCs was made to not restrict our search for possible paracrine factors only on growth factors and chemokines that have been classically involved in progression of VGD, but also on the potential role of secreted proteins such as ECM components or matrix remodeling enzymes 29. On the other hand, recent investigations by global proteomic profiling have disclosed new roles for ECM/matricellular vascular composition changes in early remodeling responses after injury 45. Our secretomic analysis revealed TSP-1 as the factor more robustly released in the conditioned medium by SMCs subjected to mechanical stress. The role of TSP-1, and in a more general view, of members of the Thrombospondin family, have been so far connected to various effects on SMCs and vascular cells, such as focal adhesion kinase function, ERK1/2, p38 and CD44 regulation, migration, proliferation and arterial remodelling 46, but never specifically linked to VGD. This is important, especially in the view of recent evidences showing the susceptibility of TSP-1 expression/function to mechanosensitive control in formation of aortic aneurysm 47 or in disturbed flow-dependent arterial stiffening 48. Although the objective of the present study was not to establish links between the response to mechanical cues and TSP-1 secretion by SV-SMCs, or to unveil the identity of mechanotransduction-dependent machineries controlling *TSP-1* gene expression at transcriptional level, it was remarkable to observe a sustained release of this factor specifically in the medial layer of CABG-stimulated human vessels (Figure 6, Figure S8), or in *in vivo* arterialized SV grafts (Figure 8). Together, these evidences support the hypothesis that release of TSP-1 in the vascular wall by SMCs is a damage response caused by SMCs mechanical stress linked to the altered vessel flow dynamics, and potentially activating/recruiting adventitial cells with a myofibroblast phenotype.

Inspired by the vision of a mechano-paracrine mechanism involving TSP-1 in SV remodeling process, we finally established a mechanism for activation of adventitial cells in SV graft pathophysiologic process. Even if these cells have been extensively characterized for their vascular regeneration potential 20, the expression of several markers in common with mesenchymal cells such as CD105, CD90 and CD44 (Figure S9) suggests a specific vascular pathophysiologic role 38. In our cell migration setting we observed a chemotactic effect of TSP-1 on SVPs. This effect was specific and involved CD47, one of the common TSP-1 receptor, expressed at high levels in these cells (Figure 7). Furthermore, compared to the treatment with the single factors, the combined treatment with TSP-1 and TGF-β increased SVPs maturation toward a SMC/myofibroblast phenotype, as shown by results of *TAGLN1*, *TGFBR1* and *Col1A* genes expression, SM22 protein expression and proliferation (Figure 7, Figure S10-S11). Taken together, these results support a multiple role of TSP-1 in activating myofibroblast-like progenitors migration from adventitial *vasa vasorum* and in modulating the latent TGF-β signaling, to promote maturation and proliferation of these cells in the media 49. This hypothesis is in line with previous observations performed in mesenchymal progenitors 30 and SMCs 46, and establishes a new function of TSP-1 in early mechanical-dependent modification of matricellular composition in human SV grafts. The potential of our mechano-dependent graft pathology model was finally tested in the SV into carotid interposition in pigs, a widely accepted, robust and reproducible large animal system to assess vein arterialization. Data obtained in this system (Figure 8) confirmed the overexpression of TSP-1 and in the vascular wall of transplanted SVs and a similar dynamics of SM22α/CD44^+^ in the adventitial region, and this correlated with neointima accumulation.

In conclusion, our study unravels for the first time a molecular mechanism linking mechanical injury occurring in coronary SV grafts with programming of intima hyperplasia by a mechano-paracrine effect. While this evidence demonstrates the relevance of cell-based mechanosensing in fibrotic diseases of the cardiovascular system, it calls for more focused studies addressing the potential reduction of intima thickening, e.g. by treatments with peptides inhibiting the TSP-1 function to resolve the timely issue of venous CABGs occlusion 50.

## Supplementary Material

Supplementary methods, figures, and tables.
