# Supplementary material for: Coronary artery mechanics induces human saphenous vein remodelling via recruitment of adventitial myofibroblast-like cells mediated by Thrombospondin-1
Source: Theranostics. 2020 Feb 3;10(6):2597–611. doi: 10.7150/thno.40595 (PMC7052885; doi:10.7150/thno.40595)
Supplement: Supplementary file 1 — Supplementary methods, figures, and tables. [file thnov10p2597s1.pdf]

Coronary artery mechanics induces human saphenous vein remodeling *via* recruitment of adventitial myofibroblast-like cells mediated by Thrombospondin-1

Gloria Garoffolo[1, 2, †], Matthijs S. Ruiter[1,†], Marco Piola[3] , Maura Brioschi[4], Anita C. Thomas[5], Marco Agrifoglio[6], Gianluca Polvani[6], Lorenzo Coppadoro[3], Gaia Spinetti[7], Stefano Zoli[8], Claudio Saccu[8], Cristina Banfi[4], Gianfranco B. Fiore[3], Paolo Madeddu[5], Monica Soncini[3] and Maurizio Pesce[1,*]

Supplementary Material

Supplementary Methods

Ex vivo SV tissue stimulation

For bioreactor-based *ex vivo* stimulations, we employed exclusively SV segments obtained from patients undergoing CABG intervention (Table S1). To minimize the damage due to vessel manipulation in the surgery theatre, SVs employed for CABG implantation were harvested using a ‘no touch’ procedure[1, 2], which maintains almost integrally the adventitia. This is a standard procedure adopted by our cardiac surgery Teams to maintain patency in implanted SV grafts and maximize the clinical outcome. In order to maximize the vitality of the SV, the vessels were maintained hydrated in saline solution for the whole duration of the surgery. After vessels were released from the surgery room, they were stored in DMEM supplemented with 10% FBS, 1% L-Glutamine (L-Glut), 1% P/S (all by Lonza) at 4 °C until *ex vivo* tissue stimulation into bioreactors. As a quality checking process, they were measured in length and calibre at the two extremities. Indeed a variable calibre and length of the vessels may result into significant variability in the flow/pressure patterns experienced especially in the CABG. In any instance, SVs shorter than 5 cm and with a calibre < 5 mm were excluded from the study. Additionally, to avoid confounding boundary effects, the parts of the SVs considered to analyse the effect of mechanical forces were derived from the central portions (~3 cm in length) while the two extremities that were anchored to inlet/outlet of the stimulation systems by vessel loops were always discarded. SV segments were mounted in an EVCS mimicking coronary hemodynamics (CABG-like stimulation, luminal pressure: 80 - 120 mmHg; pulse frequency: 1 Hz; mean flow rate: ~150 mL/min)[3] or venous conditions as controls (VP, continuous luminal pressure: 5 mmHg; flow rate: 5 mL/min)[4, 5] and cultured for 7 or 14 days. DMEM supplemented with 10% FBS, 1% L-Glut, 1% P/S, and containing 3.5% Dextran (450000-650000 kDa, Sigma-Aldrich) was used to mimic blood-like viscosity of 3.2 cP in CABG-like stimulation. Medium (1/2 of the volume) was changed every 4 days. At the end of the stimulation period, SV samples were recovered. Portions of the stimulated SV were in part processed for histology and immunofluorescence and in part for proteomic analyses. Untreated tissue rings of each SV sample were fixed and snap-frozen upon arrival from the surgery theatre and stored to serve as baseline controls. In addition, to validate our venous control

condition, we determined intima thickness after conventional passive culture of SV segments, a common model of intimal hyperplasia described before[6-8], in which rings are cultured in medium (DMEM supplemented with 10% FBS, 1% L-Glut, 1% P/S) in the absence of mechanical stimuli.

Tissue morphometry and immunohistochemistry

Fixed tissue rings were embedded in paraffin and cut at 5 μm using a rotary microtome (Leica). Lumen perimeter and the distance of the outer part of the media were measured on sections stained with Masson's trichrome (MT) staining. Intima thickness was measured on sections stained with Weigert van Gieson (WvG, both from Bio-Optica Milano, Italy), and media thickness was determined as the difference between the two measurements. Digital images were acquired using a light microscope and dedicated software (AxioVision Bio Software, Carl Zeiss, Germany). For immunohistochemistry, after heat-induced epitope unmasking (citrate buffer, pH 6, 10 min) and quenching with hydrogen peroxide (0.6%, 20 min), nonspecific binding was blocked with bovine serum albumin (BSA 3%, 45 minutes, Sigma-Aldrich) and sections were incubated overnight with a primary antibody against TSP-1 (mouse anti-human A6.1, 2 μg/mL, Invitrogen). Subsequently, sections were incubated with a secondary antibody (rabbit anti-mouse IgG HRP, Invitrogen) for 1 h, after which colour was developed with diaminobenzidine (ImmPACT DAB, DBA, Italy) and nuclei were counterstained with hematoxylin. Apoptosis was determined on sections stained with the Deadend Colorimetric TUNEL System (Promega, Italy) according to the manufacturer's protocol and with a hematoxylin counterstain.

Tissue and Cell Immunofluorescence

Immunofluorescence (IF) staining for different markers was performed on sections after epitope unmasking and blocking with BSA (3%, 1 h). Sections were incubated overnight at 4 ˚C with primary antibodies and subsequently with appropriate AlexaFluor-conjugated secondary antibodies (Invitrogen) for 1 h at RT (Table S3). Cells positive for the different markers, counted in at least 3 fields per section, were expressed as percentage of total cells in the media. For cellular IF, after fixation with 4% paraformaldehyde (Sigma-Aldrich), cells were permeabilized for 30 min at RT with PBS containing 3% (w/v) BSA and 0.2% (v/v) Triton X-100 (AppliChem), followed by primary antibody incubation at 4 °C overnight. Negative control cells were incubated in a PBS solution containing 3% BSA. AlexaFluor labelled secondary antibodies were employed to detect primary antibodies. Digital images were obtained using an ApoTome fluorescence microscope or LSM-710 confocal scanning microscope (both Carl Zeiss, Germany). All measurements and quantifications were performed using ImageJ (version 1.46r, National Institutes of Health, USA). Analysis of 3 fields per section was found to be a good representation of markers expression in the whole tissue, based on the magnification used (10 X), and the average size of the sections. Four sections representative of four consecutive portions of the stimulated vessels were quantified for each condition and for each marker. Positive cells were counted in image files blinding the type of stimulation to the examiner. Marker^{+} cells were generally scored just when they clearly exhibited a complete staining around (membrane markers) or inside cytoplasm (cytoskeleton) or nuclei (proliferation markers). This staining pattern was visible only in tissue sections stained with the

primary/secondary antibody combinations and not in control staining that were included in every set of immunofluorescence labelling.

Isolation and culture of SV-derived cells

SVPs and SMCs for in vitro experiments were isolated from SVs of patients subjected to unilateral saphenectomy (Table S1). SVP isolation was performed as described previously [9]. In brief, the vein was mechanically minced and digested for 4 h at 37 °C with 3.7 mg/mL Liberase 2 (Roche). Remaining aggregates were removed through filtration with 70 µm and 40 µm cell strainer. $CD34^{POS}/CD31^{NEG}$ cells were isolated by magnetic bead-assisted cell sorting (MACS, Miltenyi Biotec). Cells were grown in a humidified atmosphere (95% air, 5% CO2) at 37 °C in Endothelial Growth medium (EGM-2) supplemented with 2% Fetal Bovine Serum (FBS) and 1% Penicillin/Streptomycin (P/S, all by Lonza). For SMCs isolation, after removing the external tissues, including the adventitia, veins were cut longitudinally to remove the endothelium by gentle scraping and then finally minced. Tissue fragments were incubated in Dulbecco Modified Eagle's Medium (DMEM) containing Liberase for enzymatic digestion. The resulting cells were cultured in DMEM supplemented with 10% FBS and 1% P/S. Passages between 4 and 6 were used for the experiments.

SVPs flow cytometry characterization

SV-derived progenitor cells were characterized by flow cytometry analysis. Cell suspensions were incubated with a combination of directly conjugated antibodies against CD31, CD36, CD44, CD47, CD90, CD105, CD140b and NG2 (all from BD Bioscience) for 15 minutes at room temperature (RT) in the dark. Data were acquired with FACSAria flow cytometry (BD Biosciences) and analyzed with flow cytometric sorting Diva software (BD Biosciences).

In silico modelling of strain responses in SV media and adventitia

An in-silico model was implemented with the aim of quantifying the deformation experienced by the cells embedded in different extracellular matrices, namely the adventitial layer or the medial layer. The cell was assumed as a 10 µm-diameter sphere embedded in an ECM volume. The cell and the ECM perfectly adhere at their interface. A 40 µm-edge cubic ECM volume was modelled around the cell, with two additional lateral extension volumes that were used to smooth the edge effects caused by the application of lateral displacements as the mechanical loading condition. The overall volume (ECM plus cell) was meshed with about ~ 230.000 tetrahedral elements. A linear elastic constitutive model was used for the materials representing the ECM and the cell. Young's moduli were set according to the literature: Young's moduli were set according to literature: 1 kPa for cells [10] 2 kPa for the adventitia (which was treated as a soft collagen matrix [11]) and 88 kPa for the media layer [12, 13]. Lateral displacements were applied to the lateral surfaces of the extension volumes, in such a way as to simulate a 16% strain for the cubic ECM volume. Such strain value was meant to represent the circumferential strain at which the vessel matrix is subjected when it is loaded with an arterial-like pressure. It was estimated by means of the Laplace law, considering blood pressure in the range 80-120 mmHg, a vessel diameter of 2.5-3 mm, a media thickness of 0.52 mm (as measured from histological slices of 9 SV samples),

and the media Young modulus of 88 kPa, under the assumption that the pressure load is supported entirely by the medial layer.

In vitro cell straining

To investigate the effect of isolated mechanical strain on cultured cells, SMCs were subjected to cyclic strain using the FlexCell Tension Plus FX-5000T system (Flexcell International Corp., Hillsborough, NC). Before cell seeding, six-well Uniflex plates were surface-coated with bovine fibronectin (10 µg/mL; Sigma- Aldrich) in PBS after covalent crosslinking with a crosslinking reagent (sulfosuccinimidyl 6-(4'-azido-2'-nitrophenylamino) hexanoate; Sulfo-SANPAH; Pierce) at 0.2 mg/mL in Hepes 50 mM (pH 8.5), photo-activated by exposure to UV-light (365 nm). Cells were seeded 10^5/well and allowed to attach overnight before beginning the uniaxial cyclic deformation protocol (0-10% deformation, 1 Hz frequency), for 24 and 72 h. Static controls were provided by seeding an equal amount of cells into the same FN-coated plates, keeping them under the same atmospheric conditions but without mechanical stimulation.

Western and ELISA analyses

Western blot analyses were performed according to standard procedures. Cells were lysed in a buffer containing 10 mM Tris-Cl, pH 7.4, 150 mM NaCl, 5 mM EDTA, 1% (v/v) Triton X-100, 1% (w/v) sodium deoxycholate, 0.1% (w/v) sodium dodecyl sulfate and 1% (v/v) protease and phosphatase inhibitor mixture (Sigma-Aldrich). Whole cell lysates were sonicated, centrifuged for 15 min at 14 000 g; cell supernatants were then collected. Proteins were quantified by BCA protein assay kit (Pierce Chemical Co). Cell lysates (30 µg per lane) were diluted in Laemli sample buffer, heated at 95 °C for 5 min, run onto 4-12% gradient SDS-polyacrylamide gels (Invitrogen), and transferred to nitrocellulose membranes. The blots were blocked with Tris Buffered-saline containing 5% (w/v) nonfat dried milk (AppliChem) at RT for 1 h. Overnight incubation at 4 °C with primary antibodies listed in Table S4 was performed to examine individual protein expression. Membranes were finally incubated with appropriate secondary antibodies for 20 min. Images were taken by LI-COR Odyssey and band densities were quantified using ImageJ software. An enzyme-linked immunosorbent assay (ELISA, #BMS2100, Invitrogen) was performed on conditioned medium of strained *vs.* control SMCs according to the manufacturer's instructions to detect the levels of human TSP-1. Calibration curves were prepared using purified standards for the protein assessed and curve fitting was accomplished by regression following the manufacturer's instructions.

mRNA analysis

Total RNA was extracted from cell lines using TRIzol (Invitrogen), quantified with NanoDrop-1000 spectrophotometer (Thermo Fisher Scientific) before integrity assessment with an Agilent 2100 Bioanalyzer (Agilent Technologies). Superscript III (Thermo Fisher Scientific) was used for reverse transcription. Quantitative real-time PCR analysis were performed with Power SYBR Green PCR Master Mix (Applied Biosystems) in an ABI 7900 Fast thermal cycler to detect *TSP-1*, *TAGLN*, *TGFβR*, *Coll1A* gene amplification products (primers details in Table S5). The reported expression levels were calculated relative to GAPDH

mRNA, used as an internal standard control. The fold change of the genes in strained condition vs. control samples was calculated as $2^{-\Delta\Delta CT}$ and the statistical analysis was done on the ΔCT values.

Mass spectrometry of culture supernatants

For the analysis of cell secretome by label-free mass spectrometry, the conditioned media were collected from strained or not-strained SMCs and processed as described [14, 15]. Quantitative label-free LC-MSE was performed on a hybrid quadrupole-time of flight mass spectrometer (Synapt-MS, Waters Corporation, Milford, USA) as previously described [16, 17]. The proteins were identified and quantified using Progenesis QIP for proteomics (v3.0, NonLinear dynamics, Newcastle upon Tyne, UK) with a human species-specific UniProt database (release 2017.1; 20,201 entries). For SVPs migration with static/dynamically cultured SMCs conditioned medium, the culture supernatant of 3 SMCs independent donors was pooled after ELISA test checking of TSP-1 and concentrated by lyophilizing/dialyzing. TSP-1 content of the resuspended CM was checked by ELISA before performing migration experiments.

SVP Migration assay

Migration capacity was measured by Transwell assays (Corning). SVPs (15,000) were seeded in EGM-2 without FBS in the upper part of a cell culture-chamber-insert system separated from the lower chamber by an 8 µm PET membrane. In the lower compartment, four conditions were assayed: EGM-2 without FBS (negative control), EGM-2 plus 10% FBS (positive control), EGM-2 plus recombinant human TSP-1 (experimental condition 1, 10 µg/mL, R&D Systems) and EGM-2 10% FBS plus TSP-1 (experimental condition 2, 10 µg/mL), or SMCs conditioned medium. After 24 h, non-migrating cells in the upper compartment were scrapped off with a cotton swab, while cells on the lower side of the membrane were fixed with 4% paraformaldehyde for 10 min and permeabilized with methanol for 20 min at RT. Migrated cells were then stained with 1% (w/v) Crystal Violet diluted in 2% (v/v) ethanol for 30 min and images were acquired with Axiovert 200M (Zeiss). For Crystal Violet quantification, the staining was solubilized with 2% (v/v) SDS and optical density (550 nm) was measured using Infinite M200 PRO reader (Tecan). To inhibit TSP-1-dependent migration, cells were incubated at 37 °C for 30 min in serum-free EGM2 in presence or absence of function blocking antibody to CD47 (clone B6H12, 20 µg/mL, Invitrogen) and its isotype control (IgG1, 20 µg/mL, Invitrogen) before subjecting them to the transwell assay.

In vivo porcine arterialization SV model

Anaesthesia was induced with intramuscular ketamine administration (0.1 mg/Kg). After endotracheal intubation, anaesthesia was maintained using halothane, the animals ventilating spontaneously throughout. A 12-15 cm of the left long saphenous vein was isolated using a 'no touch' technique[18-21], the vein divided and stored in iso-osmotic sodium chloride solution (containing 2 IU/mL heparin (CP Pharmaceuticals Ltd, Wrexham, UK) and 50 µg/mL-glyeryl trinitrate (Schwarz Pharma, Bucks, UK; room temperature) until required, to prevent spasm. The animal was heparinized by intravenous administration of 100 IU/Kg of heparin. Both common carotid arteries were exposed via longitudinal neck incisions medial to the

sternomastoid muscle. End-to-end interposition grafts were created in both common carotid arteries (using continuous 7-0 Prolene or Surgipro), with reversed 45°-bevelled 3 cm segments of saphenous vein replacing 45°-bevelled 1 cm excised segments of carotid artery. The proximal and distal anastomoses were approximately 4 cm apart. The order of performing the grafts and the site of insertion (left or right) were randomized between procedures and the vein segments. Neck and leg wounds were closed in layers, and the animals given antibiotic cover (ampicillin (200 mg amfipen (MSD Animal Health, UK) in sterile water i.m.) and appropriate levels of analgesia (buprenorphine (Vetergesic (Ceva Animal Health Ltd, UK) 15 µg/Kg i.m.), repeated as necessary). Animals were observed continually during recovery, which always took less than 1 h. Once recovered, the animals were inspected daily and fed a normal diet and given water ad libitum. Analgesia was continued as required, but no animal required additional antibiotic cover.

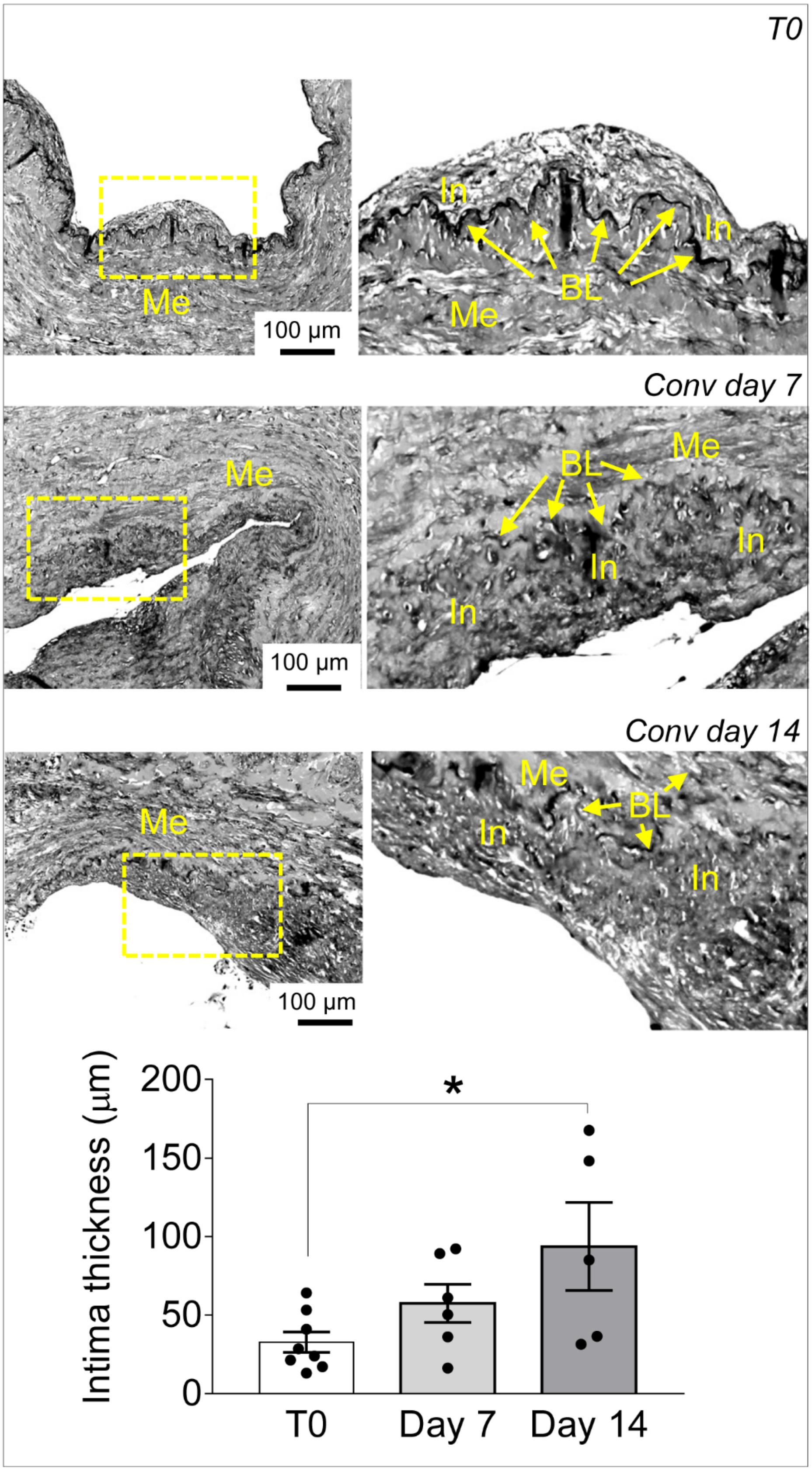

Figure S1. Low and high magnifications (zones enclosed in the yellow areas) of transversal sections of conventionally cultured SVs to show intima hyperplasia (IH) as demonstrated in the literature [22]. Yellow arrows indicate the basal lamina (BL), particularly evident in B/W pictures of Weigert van Gieson staining. Bar graph on the bottom shows quantitative evaluation of intima thickness at the indicated time points. *

indicates P < 0.05 by one-way ANOVA with Newman-Keuls multiple comparison post-hoc test. Me = Media; In = Intima. Bar graph represent mean and SE of observations.

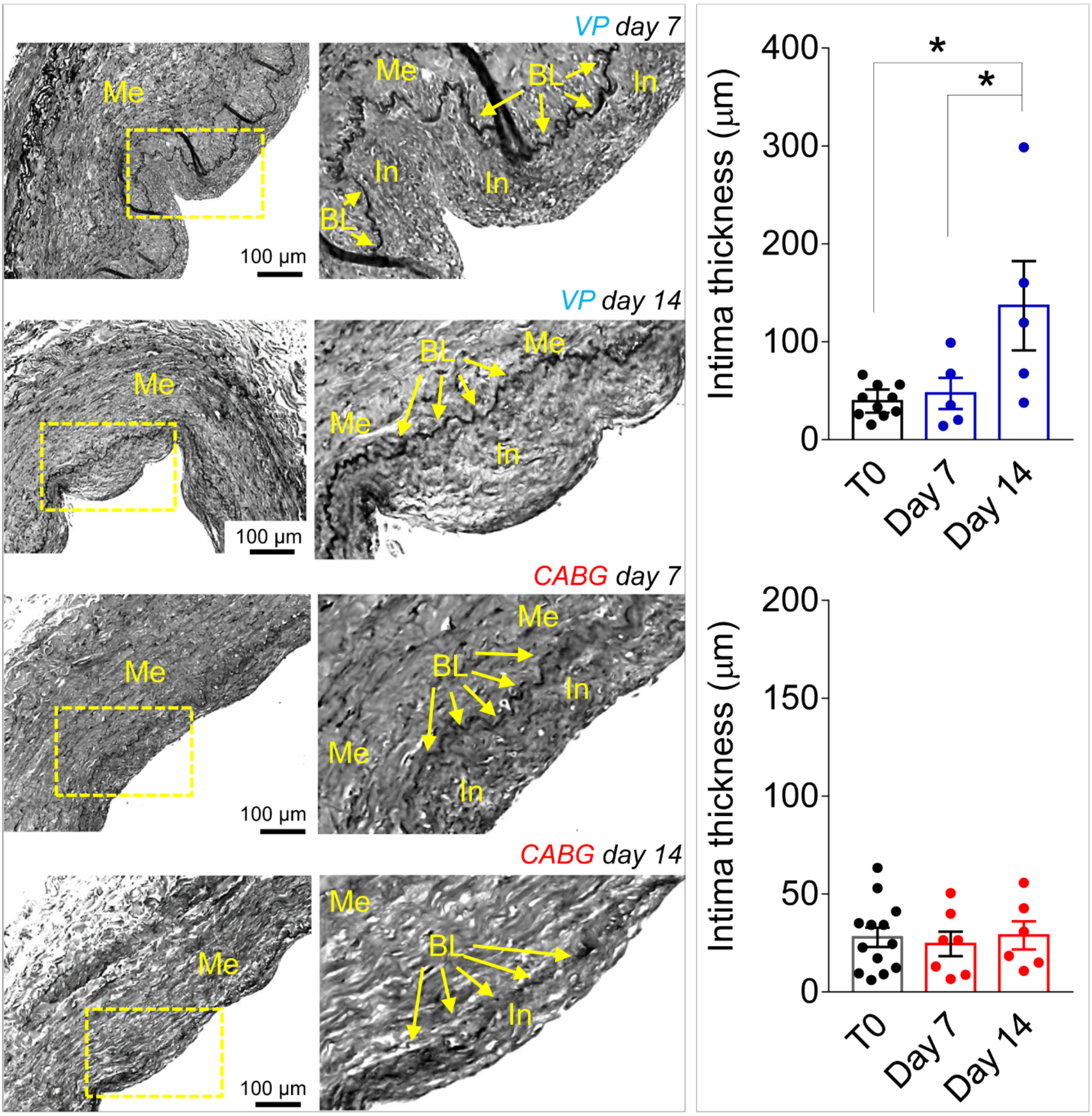

Figure S2. Low and high magnifications (zones enclosed in the yellow areas) of transversal sections of native SVs (T0) and SVs exposed to venous perfusion (VP) or coronary flow (CABG) for 7 and 14 days stained with Weigert van Gieson solution. Yellow arrows indicate the basal lamina (BL) evident in high contrast B/W pictures. Bar graphs on the right show quantitative evaluation of intima thickness at the indicated time points. * indicates $P < 0.05$ by one-way ANOVA with Newman-Keuls multiple comparison post-hoc test. Me = Media; In = Intima. Bar graphs represent mean and SE of observations.

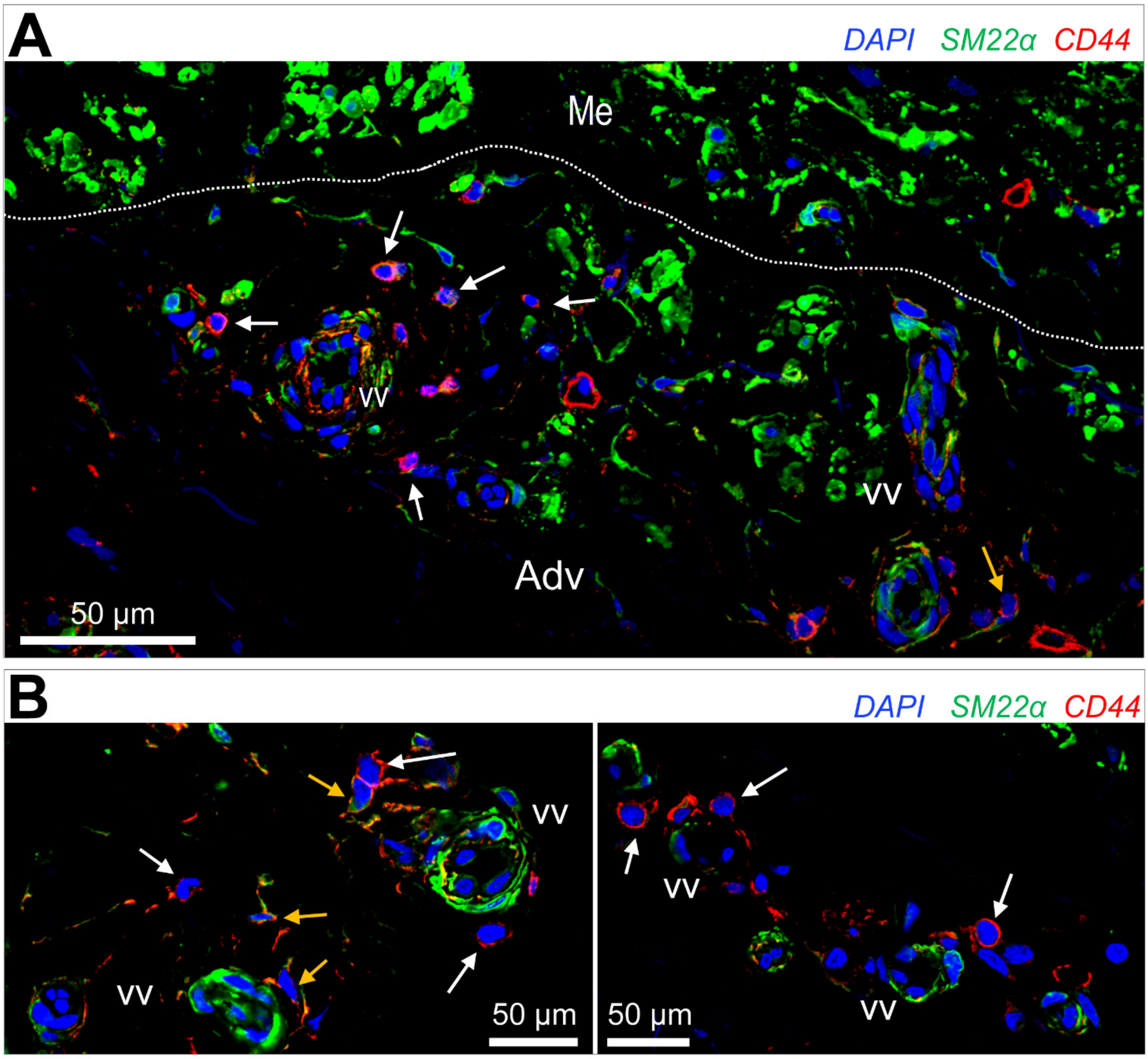

Figure S3. Low (A) and high (B) magnification of Control (T0) SV transversal sections stained with CD44 and SM22α antibodies. Note the localization of $CD44^+$ (white arrows) or $CD44^+$/$SM22\alpha^+$ cells (orange arrows) in association with the *vasa vasorum* (VV). Dotted line indicates the position of the external elastic lamina between the adventitia (Adv) and the media (Me).

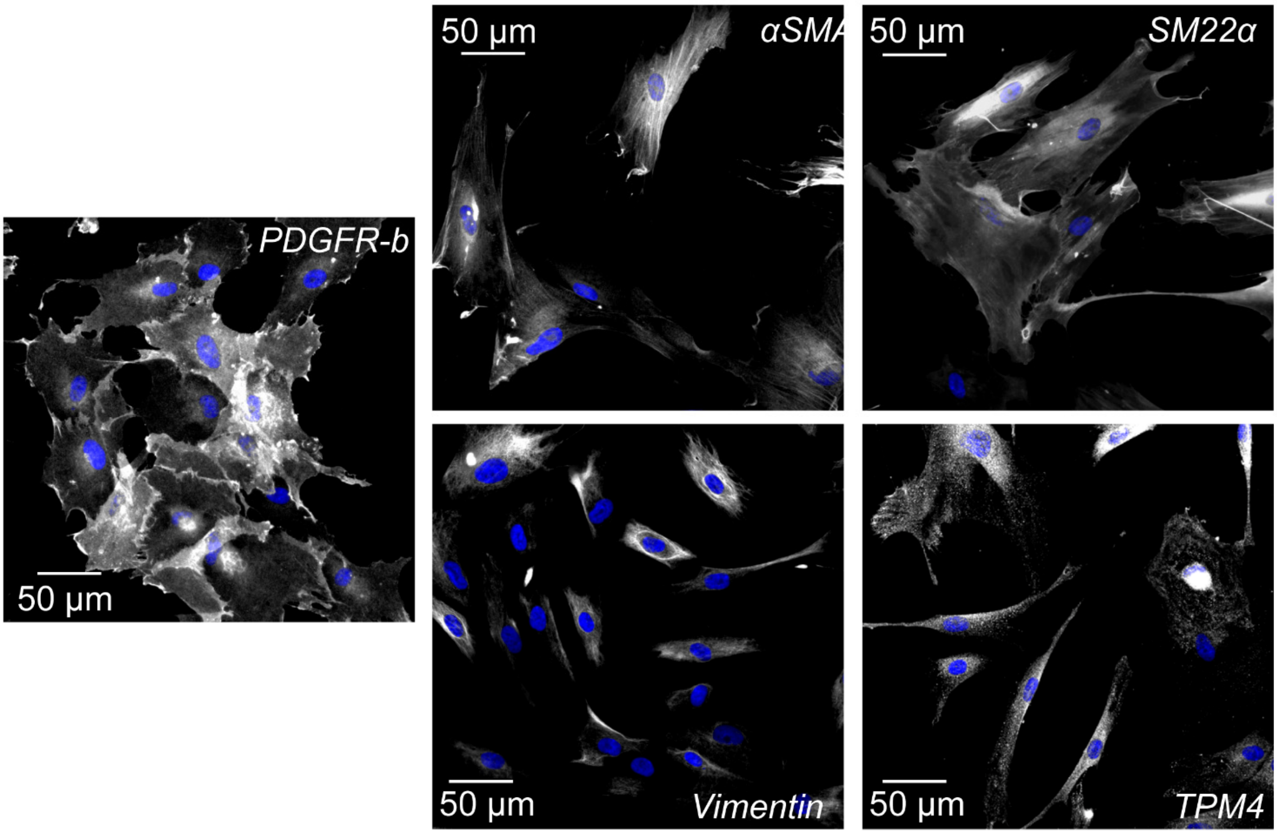

Figure S4. Characterization of SV-SMCs by immunofluorescence staining with anti PDGFR-b, αSMA, SM22α, Vimentin and Tropomyosin-4 (TPM4) specific antibodies.

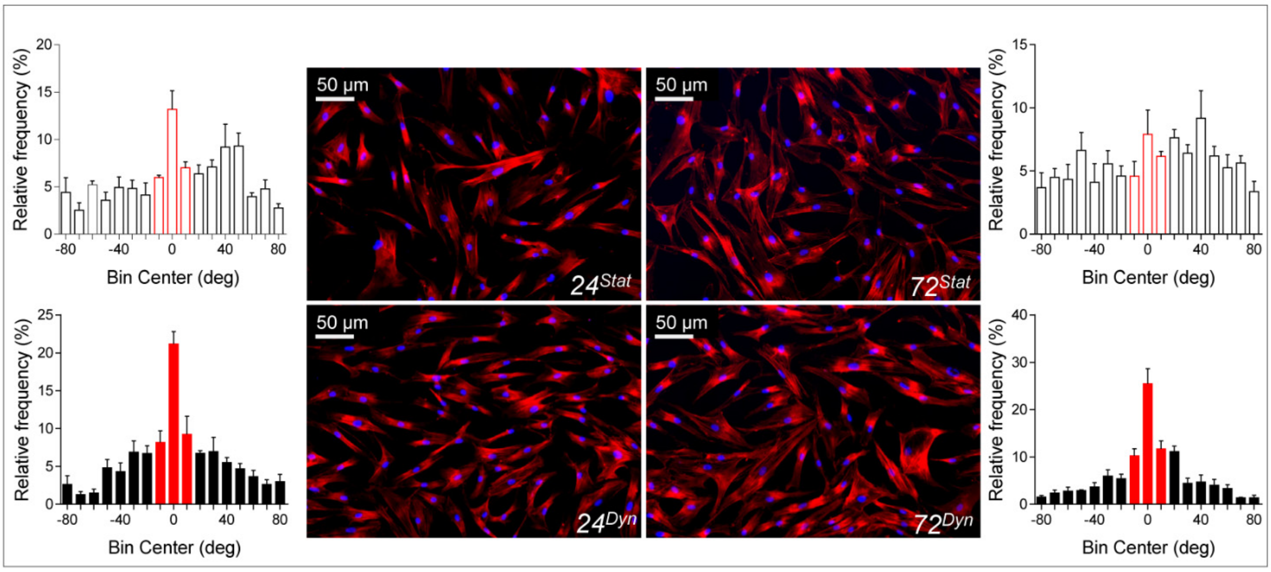

Figure S5. The panel shows the results of 24 and 72 h mechanical stimulation with a 10% uniaxial stretching regimen in the FlexCell device. The pictures in the centre show the structure of the F-actin cytoskeleton (in red, stained with Phalloidin-TRITC) in control and mechanically trained cells. The drawing in the upper part of the panel show the criterion that was adopted to assess the mechanical responses of the cells to cyclic elongation as already described by us. Namely, the frequency of the angles (θ) comprised between the major axis of the cell nuclei and an ideal orthogonal direction to the stretching were calculated and represented. From the graphical representation [23], it is evident that mechanically stimulated cells acquired a preferentially orthogonal specific orientation of their nuclei (red bars) demonstrating cytoskeleton rearrangements and mechano-sensitivity.

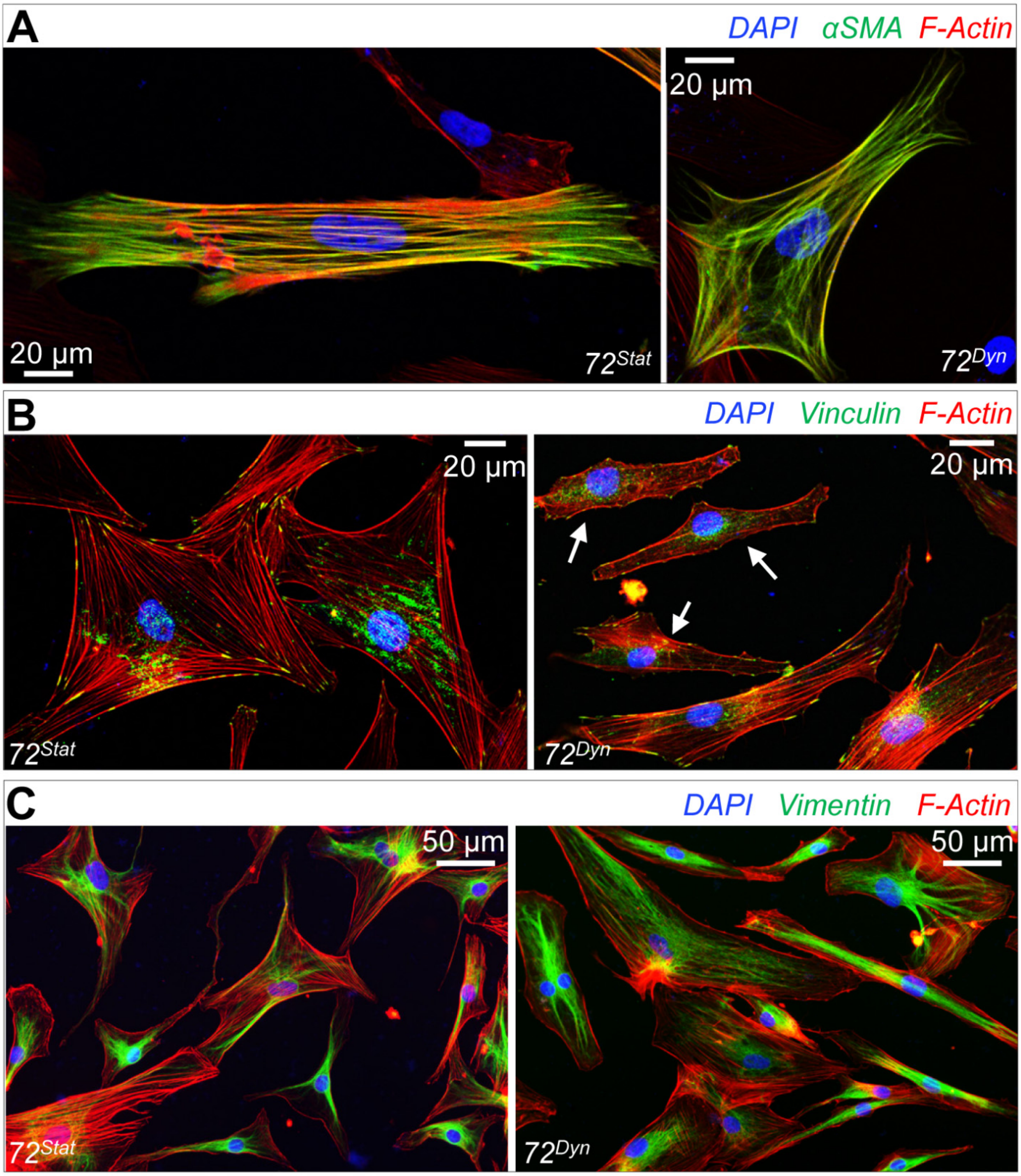

Figure S6. Immunofluorescence staining with antibodies for contractile/secretory phenotype in SV-derived SMCs subjected to mechanical strain for 72 h. In panel A, a double staining with Phalloidin-TRITC (red) and αSMA antibody (green) is shown in control cells and cells subjected to strain. Panel B represents the distribution of the focal adhesion contacts, as detected with Vinculin antibody (green dots connected to the F-actin cytoskeleton, red); arrows indicate cells with a lower organization of the F-Actin cytoskeleton and a reduced number of focal contacts. Panel C shows the expression and the intracellular distribution of Vimentin (green fluorescence). In keeping with previous data [24] and biochemical results shown in Figure 6B, the lower polymerization and organization of the αSMA^+ fibres (A), the reduced number of focal contacts (B) and the higher presence of Vimentin (C), these staining confirm the phenotypical transition between the contractile (static) and the secretory (dynamic) phenotype of SV-SMCs.

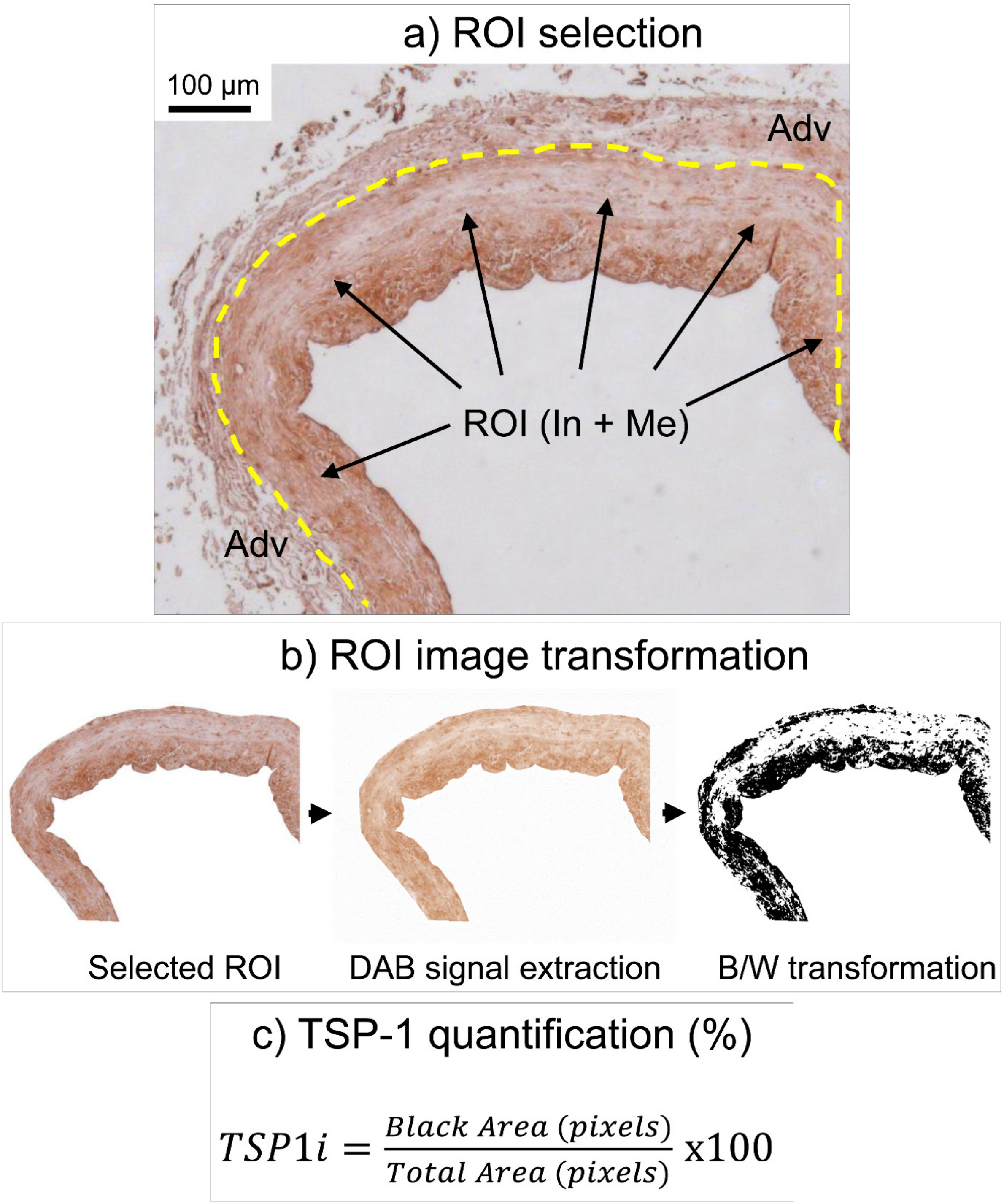

Figure S7. The upper picture shows the region of interest (ROI) in the vessel (comprising the media and the intima layers) where TSP-1 was quantified (see also **Figure 6D**). The lower panels show the Image J-based system used to quantify the expression of TSP-1 in the tissue. In order to make possible statistical comparisons (**Figure 6D**), the area (TSP-1^{+} area) occupied by black pixels was quantified and expressed as a percentage of the total area.

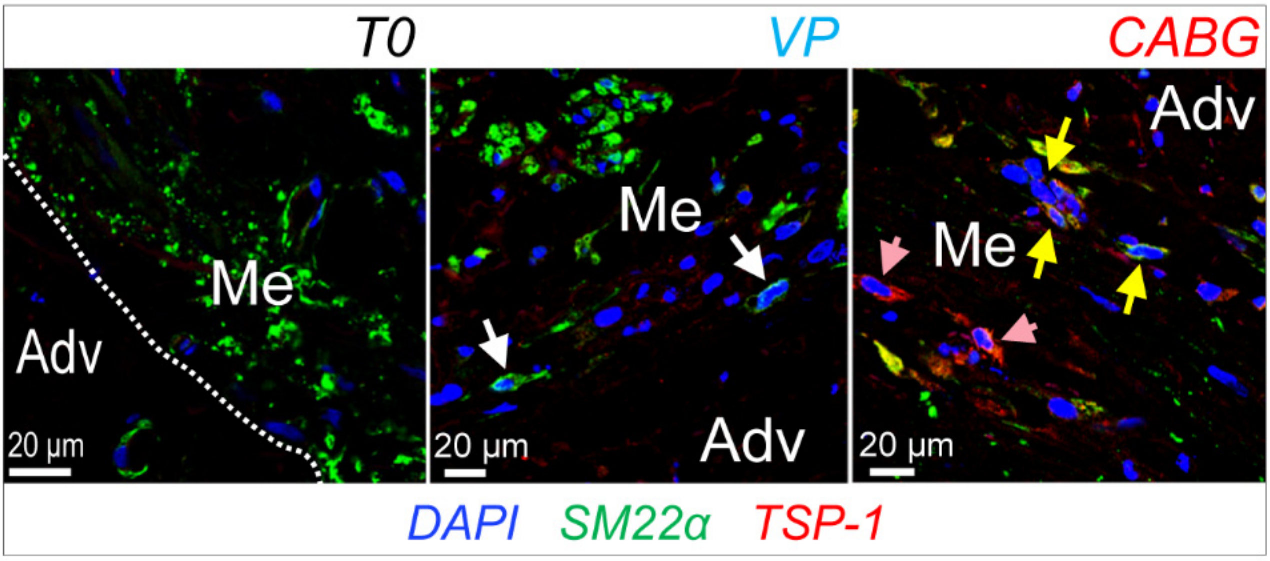

Figure S8. Immunofluorescence staining with antibodies recognizing SM22α (green fluorescence) and TSP-1 (red fluorescence) in SV samples before (T0) and after culture under venous (VP) or coronary (CABG) flow/pressure pattern for 14 days. It is evident that coronary flow mechanics increased expression of TSP-1 in cells expressing (yellow arrows) or not expressing (rose arrows) SM22α in the SV media. White arrows indicate SM22α^{+} in the picture showing the media of VP-treated SV sample.

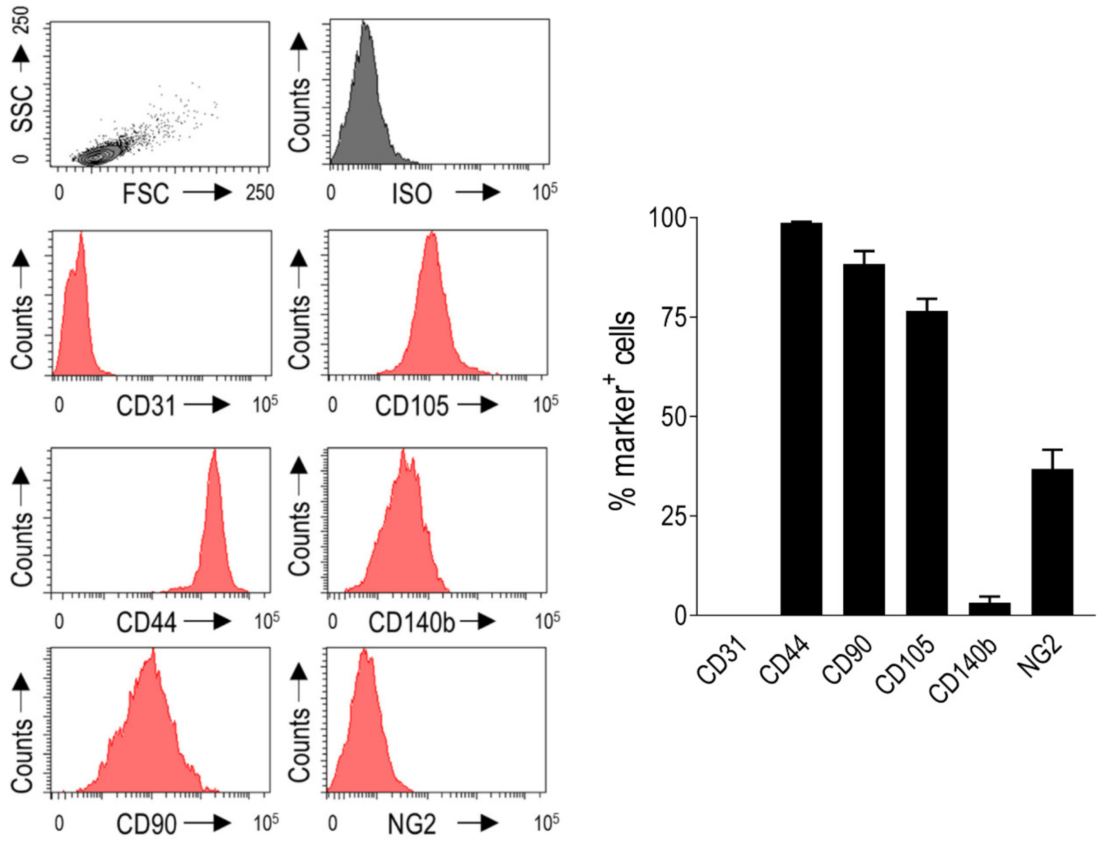

Figure S9. Characterization of human SVPs. Cells were culture amplified as described in methodological section and in reference publications[9, 25], exploiting their $CD34^{+}/CD31^{-}$ antigenic repertoire. As shown in the FACS histogram plots, SVPs expanded in culture expressed high levels of mesenchymal markers CD90, CD105 and CD44. NG2, a pericyte marker characterising cells in the SV *vasa vasorum* was, at least in part, downregulated. Bar graph represents average expression level and the relative SE of each marker in n = 37 SVPs preparations.

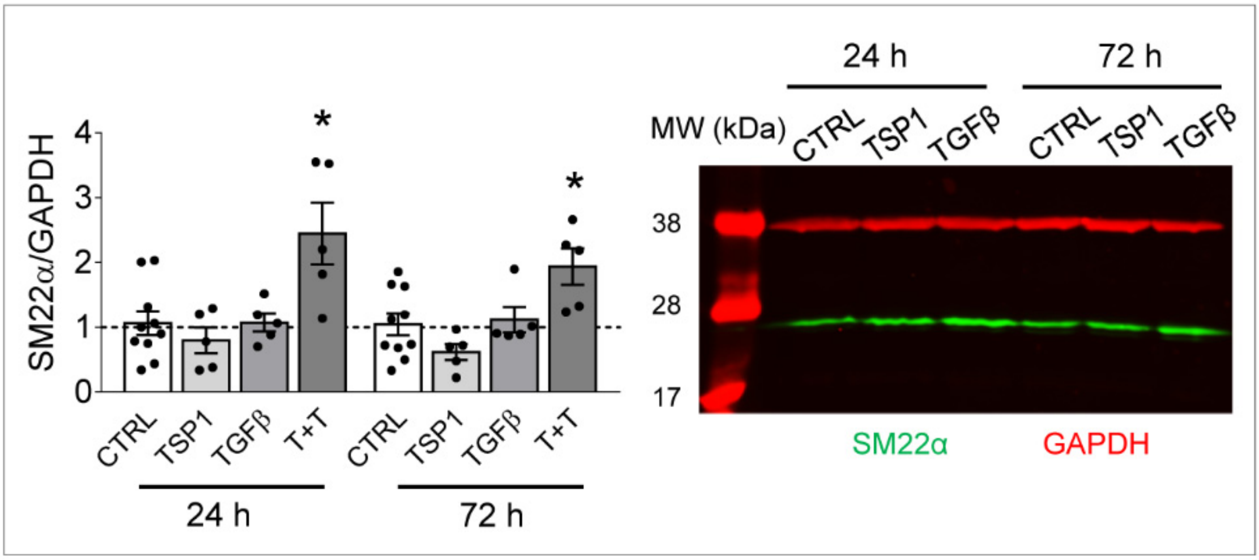

Figure S10. Effect of TSP-1 and TGF-β treatment on variations of SM22α expression in SVPs exposed to single or combined treatment (See also **Figure 7E**). * indicate $P < 0.05$ by one-way ANOVA with Newman-Keuls's comparison test. Bar graph represents mean and SE.

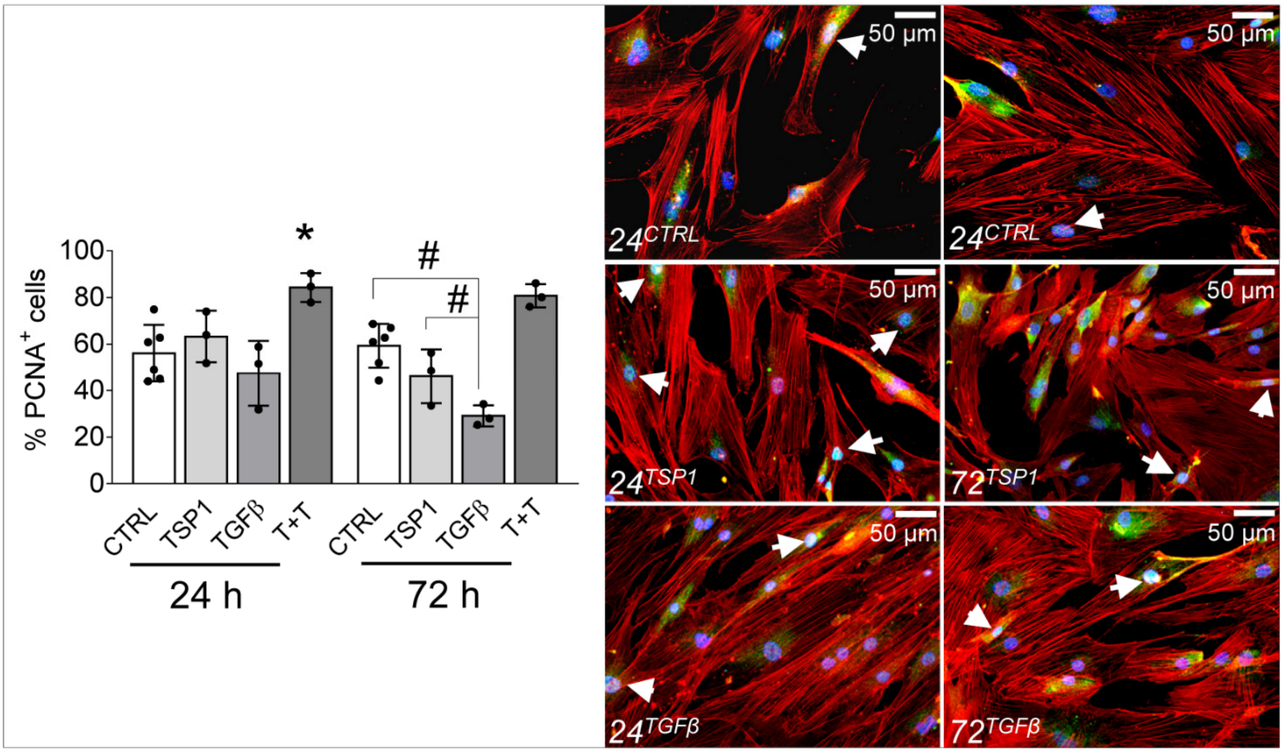

Figure S11. Effect of single/combined TSP-1/TGF-β treatment on variations in SVPs proliferation (**Figure 7F**). * indicate a statistically significant difference ($P < 0.05$) in the percentage of the PCNA^{+} cells in cells treated with TSP-1 + TGF-β *vs.* all the other treatments at both time points. # indicate a statistically significant difference ($P < 0.05$) in the percentage of the PCNA^{+} cells in TGF-β treatment *vs.* CTRL and TSP-1 treatment. Statistical analysis was performed by one-way ANOVA with Newman-Keuls multiple comparison post-*hoc* test. Bar graphs represent mean and SE of observations.

Variable	Saphenectomy patients (n = 66)	CABG patients (n = 21)
Age (y)	56 ± 13	68 ± 2
Male, n (%)	35 (53%)	19 (90%)
Hypertension, n (%)	16 (24.2%)	17 (94.4%)
Dyslipidaemia, n (%)	8 (12.1%)	10 (62.5%)
Diabetes, n (%)	3 (4.5%)	6 (35.3%)
Body mass index	26.5 ± 4.3	26.9 ± 0.6
Smoking, n (%)	22 (33.3%)	9 (42.9%)
Glycaemia	102 ± 25	
Creatinine	0.82 ± 0.2	

Table S1. Characteristics of the donors of cells (saphenectomy) and tissues (CABG surgery) collected for the study. Data are presented as percentages or mean ± SD.

24h stimulation

Description	t-test	FC (ON/OFF)	Peptide count	Unique peptides	Confidence score
**Thrombospondin-1**	**0.039**	**1.31**	**5**	**5**	**26.4**
**Cathepsin L1**	0.062	0.89	1	1	6.3
**Glia-derived nexin**	0.076	0.66	4	4	23.8
**Collagen alpha-3(VI)**	0.077	0.85	8	7	37.5
**Actin_ cytoplasmic 1**	0.085	1.10	3	3	17.4
**Histone H2B type 1-K**	0.11	1.20	1	1	6.6
**Pentraxin-related protein PTX3**	0.12	1.25	2	2	12.6
**72 kDa type IV collagenase**	0.13	0.75	2	2	10.9
**Tenascin-X**	0.167	1.49	3	3	15.3
**Metalloproteinase inhibitor 1**	0.19	0.64	3	3	20.4
**Collagen alpha-1(III) chain**	0.23	0.90	6	6	30.5
**Transforming growth factor-beta-induced protein ig-h3**	0.26	0.83	3	3	18.2
**Galectin-3-binding protein**	0.28	0.94	1	1	5.6
**SPARC OS=Homo sapiens**	0.30	1.11	3	3	16.8
**Collagen alpha-1(VI) chain**	0.37	1.09	6	6	31.2
**Fibronectin**	0.42	0.95	53	52	432.4
**Collagen alpha-1(I) chain**	0.45	0.91	29	7	194.3
**Collagen alpha-1(II) chain**	0.52	0.95	6	2	26.9
**Decorin**	0.61	1.03	6	6	32.9
**Vimentin**	0.67	1.06	6	6	32.1
**Collagen alpha-2(I) chain**	0.74	0.95	32	13	212.2
**Collagen alpha-2(VI) chain**	0.93	1.02	1	1	4.9

72h stimulation

Description	t-test	FC (ON/OFF)	Peptide count	Unique peptides	Confidence score
**Collagen alpha-1(VI) chain**	**0.009**	**0.82**	**24**	**23**	**195.6**
**Pigment epithelium-derived factor**	**0.013**	**0.87**	**6**	**6**	**37.7**
**Thrombospondin-1**	**0.029**	**1.99**	**21**	**20**	**154.8**
**SPARC**	**0.030**	**1.20**	**6**	**6**	**48.7**
**Fibronectin**	**0.042**	**0.68**	**79**	**37**	**779.8**
**Cartilage oligomeric matrix protein**	**0.043**	**0.82**	**7**	**7**	**69.1**
Collagen alpha-3(VI) chain	0.05	0.71	22	20	122.3
Target of Nesh-SH3	0.079	0.70	5	5	34.3
Decorin	0.091	0.77	11	9	71.2
Fibrillin-1	0.095	0.86	4	4	24.8
Collagen alpha-1(III) chain	0.12	0.83	44	35	332.4
Collagen alpha-2(VI) chain	0.13	0.90	14	12	82.5
Collagen alpha-1(I) chain	0.15	1.14	75	11	621.6
72 kDa type IV collagenase	0.19	0.84	6	6	42.7
Transforming growth factor-beta-induced protein ig-h3	0.19	0.71	8	8	49.1
Complement C1s subcomponent	0.24	0.87	6	6	38.8
Metalloproteinase inhibitor 2	0.28	0.77	2	2	18.8
L-lactate dehydrogenase A chain	0.28	0.92	6	5	36.9
Biglycan	0.41	1.20	6	4	42.1
Vimentin	0.41	1.09	19	18	125.8
Lumican	0.44	0.91	9	9	75.0
Galectin-3-binding protein	0.44	0.87	6	5	39.4
Lactadherin	0.59	0.93	4	3	23.0
Metalloproteinase inhibitor 1	0.59	0.94	6	6	48.2
Glia-derived nexin	0.73	1.08	11	11	75.6
Clusterin	0.74	1.04	9	9	71.0
Pentraxin-related protein PTX3	0.75	1.04	9	9	64.2
Follistatin-related protein 1	0.79	1.02	5	5	28.3
Collagen alpha-2(I) chain	0.87	1.01	67	34	584.3
Collagen alpha-1(XII) chain	0.92	1.01	28	25	172.8
Insulin-like growth factor-binding protein 7	0.93	0.99	8	7	62.4
Tenascin-X	0.95	0.99	29	27	171.9

Tables S2. Results of mass spectrometry-based secretome analysis on SMCs stimulated mechanically for 24 (n = 3) and 72 (n = 6) h. In red and green colors, respectively, are indicated secreted proteins with a significantly (P < 0.05; paired t-test) increased or decreased secretion in conditioned medium. Note the presence of Thrombospondin-1 as the unique factor secreted in higher amounts in mechanically stimulated SMCs *vs.* Controls at both time points.

Antibody/ Reagent	Host	Manufacturer	Clone, product code	Concentration tissue/cells
αSMA	Mouse	Dako	1A4, M0851	142 ng/mL tissue, 700 ng/mL cells
Calponin	Mouse	Abcam	PC10, ab65827	500 ng/mL tissue
CD44	Rat	Abcam	IM7, ab119863	20 μg/mL tissue
SM22α	Rabbit	Abcam	Polyclonal, ab14106	1.4 μg/mL tissue, 8 μg/mL cells
PCNA	Mouse	Dako	PC10, M0879	1.64 μg/mL tissue, 3.27 μg/mL cells
TSP-1	Mouse	Invitrogen	A6.1, MA5-13398	2 μg/mL tissue
TPM4	Mouse	Bio-Rad	4A4-1D2, MCA5263Z	5 μg/mL
Vimentin	Rabbit	Cell signaling technology	D21H3, #5741	Diluted as indicated
PDGFRβ	Rabbit	Cell Signaling Technology	28E1, #3169	Diluted as indicated
Vinculin	Rabbit	Invitrogen	42H89L44	5 μg/mL
Phalloidin	-	Sigma Aldrich	P1951	32 μg/mL
DAPI	-	Dako	D1306	50 μg/mL
488 mouse	Donkey	Invitrogen	A11034	4 μg/mL
488 rabbit	Goat	Invitrogen	A11034	4 μg/mL
546 mouse	Donkey	Invitrogen	A10036	4 μg/mL
546 rat	Goat	Invitrogen	A11081	4 μg/mL
546 rabbit	Goat	Invitrogen	A11010	4 μg/mL

Table S3. Reagents, primary and secondary antibodies employed for tissue and cell IF analysis.

Antibody	Host	Manufacturer	Concentration
αSMA	Mouse	Dako	7 μg/mL
Vimentin	Rabbit	Cell signaling technology	Diluted as indicated
TSP-1	Mouse	Invitrogen	200 ng/mL
SM22α	Rabbit	Abcam	1 μg/mL
GAPDH	Mouse/Rabbit	Santa-Cruz	200 ng/mL
680 anti-mouse	Donkey	LI-COR	100 ng/mL
680 anti-rabbit	Donkey	LI-COR	100 ng/mL
800 anti-mouse	Donkey	LI-COR	100 ng/mL
800 anti-rabbit	Donkey	LI-COR	100 ng/mL

Table S4. List of primary and secondary antibodies used for Western Blot.

Gene	Sequence
hTSP-1	Forward: 5’-TGAGGAGGACACTGGTAGAG-3’<br>Reverse: 5’-GGGCCTCAATGACAATTTCC-3’
hTAGLN	Forward: 5’-ACAAACTCATCTTCCTCAAGCC-3’<br>Reverse: 5’-CTTCTCATTTTCCATTCCCTTCAC-3’
hCOL1A1	Forward: 5’-GGACACAGAGGTTTCAGTGG -3’<br>Reverse: 5’-CCAGTAGCACCATCATTTCC -3’
hTGFβ-R	Forward: 5’-GCCAGTCCTAAGTCTGCAAT-3’<br>Reverse: 5’-GGTCTTGCCCATCTTCACA-3’
hGAPDH	Forward: 5’-AATCCCATCACCATCTTCCAG-3’<br>Reverse: 5’-AAATGAGCCCCAGCCTTC-3’

Table S5. PCR oligo sequences of primers employed in Q-RT-PCR analysis

Supplementary references

1. Dashwood MR, Tsui JC. ‘No-touch’ saphenous vein harvesting improves graft performance in patients undergoing coronary artery bypass surgery: A journey from bedside to bench. Vascul Pharmacol. 2013; 58: 240-50.

2. Johansson BL, Souza DS, Bodin L, Filbey D, Loesch A, Geijer H, et al. Slower progression of atherosclerosis in vein grafts harvested with 'no touch' technique compared with conventional harvesting technique in coronary artery bypass grafting: an angiographic and intravascular ultrasound study. Eur J Cardiothorac Surg. 2010; 38: 414-9.

3. Piola M, Ruiter M, Vismara R, Mastrullo V, Agrifoglio M, Zanobini M, et al. Full Mimicking of Coronary Hemodynamics for Ex-Vivo Stimulation of Human Saphenous Veins. Ann Biomed Eng. 2017; 45: 884-97.

4. Piola M, Prandi F, Bono N, Soncini M, Penza E, Agrifoglio M, et al. A compact and automated ex vivo vessel culture system for the pulsatile pressure conditioning of human saphenous veins. J Tissue Eng Regen Med. 2013.

5. Prandi F, Piola M, Soncini M, Colussi C, D'Alessandra Y, Penza E, et al. Adventitial vessel growth and progenitor cells activation in an ex vivo culture system mimicking human saphenous vein wall strain after coronary artery bypass grafting. PLoS ONE. 2015; 10: e0117409.

6. Castronuovo JJ, Smith TJ, Price RM. Validation of an in vitro model of human saphenous vein hyperplasia. J Vasc Surg. 2002; 35: 152-7.

7. Osgood MJ, Hocking KM, Voskresensky IV, Li FD, Komalavilas P, Cheung-Flynn J, et al. Surgical vein graft preparation promotes cellular dysfunction, oxidative stress, and intimal hyperplasia in human saphenous vein. J Vasc Surg. 2014; 60: 202-11.

8. Kenagy RD, Kikuchi S, Evanko SP, Ruiter MS, Piola M, Longchamp A, et al. Versican is differentially regulated in the adventitial and medial layers of human vein grafts. PLoS ONE. 2018; 13: e0204045.

9. Campagnolo P, Cesselli D, Al Haj Zen A, Beltrami AP, Krankel N, Katare R, et al. Human adult vena saphena contains perivascular progenitor cells endowed with clonogenic and proangiogenic potential. Circulation. 2010; 121: 1735-45.

10. Nikolaev NI, Müller T, Williams DJ, Liu Y. Changes in the stiffness of human mesenchymal stem cells with the progress of cell death as measured by atomic force microscopy. J Biomech. 2014; 47: 625-30.

11. Roeder BA, Kokini K, Sturgis JE, Robinson JP, Voytik-Harbin SL. Tensile Mechanical Properties of Three-Dimensional Type I Collagen Extracellular Matrices With Varied Microstructure. J Biomech Eng. 2002; 124: 214-22.

12. Goldstone RN, McCormack MC, Khan SI, Salinas HM, Meppelink A, Randolph MA, et al. Photochemical Tissue Passivation Reduces Vein Graft Intimal Hyperplasia in a Swine Model of Arteriovenous Bypass Grafting. J Am Heart Assoc. 2016; 5: e003856.

13. Szentiványi Jr M, Nádasy GL, Tóth M, Kopcsányi V, Jednákovits A, Monos E. Biomechanics of the saphenous artery and vein in spontaneous hypertension in rats. Pathophysiology. 1998; 4: 295-302.

14. Brioschi M, Lento S, Tremoli E, Banfi C. Proteomic analysis of endothelial cell secretome: A means of studying the pleiotropic effects of Hmg-CoA reductase inhibitors. J Proteomics. 2013; 78: 346-61.

15. Pontremoli M, Brioschi M, Baetta R, Ghilardi S, Banfi C. Identification of DKK-1 as a novel mediator of statin effects in human endothelial cells. Sci Rep. 2018; 8: 16671.

16. Brioschi M, Eligini S, Crisci M, Fiorelli S, Tremoli E, Colli S, et al. A mass spectrometry-based workflow for the proteomic analysis of in vitro cultured cell subsets isolated by means of laser capture microdissection. Anal Bioanal Chem. 2014; 406: 2817-25.

17. Roverso M, Brioschi M, Banfi C, Visentin S, Burlina S, Seraglia R, et al. A preliminary study on human placental tissue impaired by gestational diabetes: a comparison of gel-based versus gel-free proteomics approaches. Eur J Mass Spectrom (Chichester). 2016; 22: 71-82.

18. Angelini GD, Bryan AJ, Williams HM, Soyombo AA, Williams A, Tovey J, et al. Time-course of medial and intimal thickening in pig venous arterial grafts: relationship to endothelial injury and cholesterol accumulation. J Thorac Cardiovasc Surg. 1992; 103: 1093-103.

19. Thomas AC, Wyatt MJ, Newby AC. Reduction of early vein graft thrombosis by tissue plasminogen activator gene transfer. Thromb Haemost. 2009; 102: 145-52.

20. Vijayan V, Shukla N, Johnson JL, Gadsdon P, Angelini GD, Smith FCT, et al. Long-term reduction of medial and intimal thickening in porcine saphenous vein grafts with a polyglactin biodegradable external sheath. J Vasc Surg. 2004; 40: 1011-9.

21. Izzat MB, Mehta D, Bryan AJ, Reeves B, Newby AC, Angelini GD. Influence of external stent size on early medial and neointimal thickening in a pig model of saphenous vein bypass grafting. Circulation. 1996; 94: 1741-5.

22. McDonald RA, White KM, Wu J, Cooley BC, Robertson KE, Halliday CA, et al. miRNA-21 is dysregulated in response to vein grafting in multiple models and genetic ablation in mice attenuates neointima formation. Eur Heart J. 2013; 34: 1636-43.

23. Ugolini GS, Rasponi M, Pavesi A, Santoro R, Kamm R, Fiore GB, et al. On-chip assessment of human primary cardiac fibroblasts proliferative responses to uniaxial cyclic mechanical strain. Biotechnol Bioeng. 2016; 113: 859-69.

24. Worth NF, Rolfe BE, Song J, Campbell GR. Vascular smooth muscle cell phenotypic modulation in culture is associated with reorganisation of contractile and cytoskeletal proteins. Cell Motil Cytoskeleton. 2001; 49: 130-45.

25. Gubernator M, Slater SC, Spencer HL, Spiteri I, Sottoriva A, Riu F, et al. Epigenetic profile of human adventitial progenitor cells correlates with therapeutic outcomes in a mouse model of limb ischemia. Arterioscler Thromb Vasc Biol. 2015; 35: 675-88.
